# The MAZ-POLD1 Signaling Axis Drives Cisplatin Resistance in Bladder Cancer by Activating DNA Damage Repair

**DOI:** 10.3390/cancers18172892

**Published:** 2026-09-07

**Authors:** Biao Zhang, Hong Chang, Cheng Wang, Wei Chang, Shujun Yang, Yao Luo, Yuqiang Fu, Helin Zhang, Xuan Li, Can Li, Jianzhong Lu, Su Zhang, Panfeng Shang

**Affiliations:** 1 Department of Urology, The Second Hospital & Clinical Medical School, Lanzhou University, Lanzhou 730030, China; 2 Institute of Urology, Gansu Province Clinical Research Center for Urinary System Diseases, Lanzhou 730030, China

**Keywords:** bladder cancer, cisplatin resistance, POLD1, DNA damage repair, MAZ

## Abstract

**Background**: Although POLD1 exhibits oncogenic properties in multiple malignancies, its precise role and regulatory mechanisms in cisplatin resistance of bladder cancer (BC) remain elusive. This study aims to elucidate the functional involvement and upstream regulatory axis of POLD1 in BC chemoresistance. **Methods**: We evaluated the impact of POLD1 on chemoresistance and DNA damage repair (DDR) using public clinical databases and cisplatin-resistant cell lines, employing CCK-8, colony formation, flow cytometry, immunofluorescence, and comet assays. Protein interactions were examined via Co-IP, molecular docking, and truncation mutant analysis. ChIP-PCR and dual-luciferase reporter assays were utilized to identify the upstream transcription factor. In vivo functionality was further validated in a nude mouse xenograft model. **Results**: POLD1 was markedly upregulated in BC tissues and cisplatin-resistant BC cells, and its elevated expression was tightly associated with advanced tumor stage, high pathological grade, and poor patient prognosis. Functional experiments verified that POLD1 knockdown aggravated cisplatin-induced DNA damage and drastically sensitized BC cells to cisplatin in vitro, and attenuated tumor growth under cisplatin treatment in vivo, indicating that POLD1 is a key driver of cisplatin resistance. Mechanistically, POLD1 physically interacted with and activated ATM, thereby initiating homologous recombination (HR) repair signaling. Moreover, transcription factor MAZ directly bound the promoter region of POLD1 to transcriptionally upregulate its expression. Rescue experiments in vitro further validated that the MAZ-POLD1 axis facilitates cisplatin resistance by modulating the DDR pathway in BC. **Conclusions**: Therapeutic targeting of the POLD1-regulated DDR pathway holds great promise as an effective strategy to reverse acquired cisplatin resistance in BC.

## Simple Summary

Cisplatin resistance severely limits therapeutic benefits for bladder cancer patients. In this study, we first verified that POLD1 was markedly overexpressed in cisplatin-resistant BC tissues and cells. Functional assays demonstrated that POLD1 knockdown remarkably impaired DDR capacity in cisplatin-resistant BC cells and restored their chemosensitivity to cisplatin in vitro, indicating that POLD1 is a key driver of cisplatin resistance. Further mechanistic experiments revealed that POLD1 physically interacted with ATM to facilitate ATM phosphorylation, which further triggered HR repair signaling. In addition, the transcription factor MAZ directly bound to the promoter region of POLD1 to transcriptionally activate POLD1 expression. Together, the MAZ–POLD1 axis represents a key driver of cisplatin resistance in bladder cancer. Our findings suggest that targeting this pathway may offer a promising new strategy to overcome drug resistance and improve clinical outcomes for bladder cancer patients.

## 1. Introduction

Bladder cancer (BC) is one of the most common malignancies of the urinary tract and represents a major global health burden [1,2]. Approximately 75% of newly diagnosed cases are non-muscle-invasive bladder cancer (NMIBC), which is confined to the mucosa or lamina propria without muscularis propria invasion [3]. NMIBC is primarily managed by transurethral resection of bladder tumor (TURBT) followed by risk-adapted intravesical therapy, whereas systemic cisplatin-based chemotherapy is not routinely used [3]. In contrast, cisplatin-based systemic therapy has long played a central role in the management of muscle-invasive and advanced bladder cancer [4]. For patients with muscle-invasive or metastatic BC, cisplatin-based combination chemotherapy plays a crucial role in improving prognosis and prolonging survival [5,6]. The anti-tumor efficacy of cisplatin mainly originates from its cytotoxicity triggered by the induction of DNA double-strand breaks (DSBs) in tumor cells, whereby it suppresses tumor cell proliferation and initiates apoptotic cascades. Nevertheless, the existence of intrinsic and acquired resistance mechanisms drastically compromises chemotherapeutic efficacy [7]. Clinical studies indicate that most BC patients gradually develop cisplatin resistance during treatment, ultimately resulting in therapeutic failure, tumor recurrence, and metastasis [8,9]. Therefore, elucidating the molecular mechanisms driving cisplatin resistance in BC and identifying viable therapeutic targets are of great clinical significance for boosting cisplatin responsiveness and prolonging patient survival. The known mechanisms of cisplatin resistance primarily include abnormalities in the DDR, increased intracellular cisplatin efflux, intracellular metabolic inactivation, and disruption of apoptosis pathways. Among these, DDR abnormalities are considered one of the central mechanisms driving cisplatin resistance [7,9].

The DNA polymerase 1 (POLD1) gene is located on the q13.3-q13.4 region of human chromosome 19 [10,11]. It spans approximately 34 kb, contains 27 exons, and encodes a protein consisting of 1107 amino acids [12]. POLD1 functions as the catalytic subunit of the DNA polymerase δ (Pol δ) complex, which also includes three regulatory subunits: POLD2, POLD3, and POLD4 [13]. As the catalytic core of Polδ, POLD1 exhibits both DNA polymerase and 3′-5′ exonuclease activities, playing a key role in regulating multiple DDR pathways [12,14]. During DNA replication, POLD1, in association with proliferating cell nuclear antigen (PCNA), is responsible for synthesizing the lagging strand through its polymerase activity [15]. Additionally, its 3′-5′ exonuclease activity performs error-correction functions, significantly enhancing the accuracy of DNA replication and reducing genomic instability caused by replication errors [16]. POLD1 also helps maintain the balance between cell proliferation and apoptosis by regulating the cell cycle [17]. Recent studies have demonstrated that POLD1 can exert an oncogenic role in BC and renal clear cell carcinoma, independently of the Pol δ complex [18,19]. These findings suggest that POLD1 may contribute to tumorigenesis, tumor progression, and the development of chemotherapy resistance in ways that are independent of its role in the Polδ complex. However, the specific role and molecular mechanisms of POLD1 in cisplatin resistance in BC remain to be fully elucidated.

MYC-associated zinc finger protein (MAZ) is an important transcription factor that is widely expressed across various human tissues [20,21]. Its protein structure consists of six tandem C2H2-type zinc finger domains, which specifically recognize and bind to G/C-rich DNA sequence elements, thereby regulating the transcriptional expression of downstream target genes [22]. For instance, MAZ can positively regulate the expression of the c-MYC gene by binding to the GA box sequence (5′-GGGAGGG-3′) within the P2 promoter region of the c-MYC gene, thereby influencing tumor cell proliferation [23]. Numerous studies have demonstrated that MAZ is abnormally overexpressed in various malignant tumors, including hepatocellular carcinoma, breast cancer, prostate cancer, and neuroblastoma, where it plays a critical role in tumor progression, metastasis, and treatment resistance [24,25,26,27]. Other research has shown that MAZ can mitigate DNA damage accumulation by regulating NEIL3 gene expression, thereby promoting cisplatin resistance in lung cancer cells and enhancing their migration and invasion abilities [28]. However, the specific regulatory mechanisms by which MAZ contributes to cisplatin resistance in BC through transcriptional reprogramming and the regulation of downstream target gene expression remain unclear.

In this study, we first verified that POLD1 was markedly overexpressed in cisplatin-resistant BC tissues and cells. Functional assays demonstrated that POLD1 knockdown remarkably impaired DDR capacity in cisplatin-resistant BC cells and restored their chemosensitivity to cisplatin. At the molecular mechanistic level, we validated that POLD1 physically interacts with ATM to facilitate ATM phosphorylation, which further triggers HR repair signaling. In addition, the transcription factor MAZ physically interacts with the promoter region of POLD1 to transcriptionally activate POLD1 expression. Functional rescue experiments demonstrated that re-expression of POLD1 in MAZ knockdown cells partially restored DDR capacity and rescued cisplatin resistance. Collectively, this study delineates the molecular mechanism by which the MAZ-POLD1 axis modulates cisplatin resistance in BC, and provides a promising therapeutic target for clinical intervention against cisplatin-resistant BC.

## 2. Materials and Methods

### 2.1. Dataset Acquisition and Processing

In this study, data were integrated from public database resources and in-house sequencing datasets. Gene expression profiles and matched clinical data including GSE188715, GSE32894, GSE48075 and GSE13507 were downloaded from the Gene Expression Omnibus (GEO, https://www.ncbi.nlm.nih.gov/geo/, accessed on 10 July 2025). Meanwhile, bulk RNA-seq was performed on parental T24 cells and cisplatin-resistant T24CisR cells, and the FPKM-normalized expression matrix was provided in Supplementary Table S1. The Shiny TF-Target Finder database [29] was utilized to predict the regulatory relationship between transcription factor MAZ and POLD1, followed by correlation analysis of their expression levels. The JASPAR database (https://jaspar2022.genereg.net/, accessed on 21 February 2026) was applied to predict putative MAZ binding sites within the promoter region of the POLD1 gene.

### 2.2. Cell Culture and Chemical Reagents

Two human BC cell lines (UMUC3 and T24) and 293T were purchased from Cell Bank, Chinese Academy of Sciences. The human BC cell lines T24 and UMUC3 were routinely cultured in RPMI 1640 medium supplemented with 10% fetal bovine serum (FBS, Sigma, Burlington, MA, USA). 293T cells were cultured in DMEM medium containing 10% FBS. Cisplatin-resistant T24CisR and UMUC3CisR cell lines were generated by continuous culture with gradually elevated cisplatin concentrations over a period of six months, and maintained routinely in medium supplemented with 2 μM cisplatin to sustain their drug-resistant phenotype. Briefly, parental T24 and UMUC3 cells were continuously exposed to incrementally increasing concentrations of cisplatin starting from 0.5 μM. The concentration was elevated by 0.5 μM every 2–3 passages once cells adapted and maintained stable proliferation. After approximately 6 months of induction, the final maintenance concentration was set at 2 μM. The resistant phenotype was verified by a resistance index (IC50 of resistant cells/IC50 of parental cells) greater than 3, which meets the widely accepted criteria for successful acquisition of cisplatin resistance in bladder cancer research. All cells were cultured in a humidified incubator at 37 °C with 5% CO_2_, and medium changes were performed regularly to maintain optimal cell growth conditions. The cell lines were provided by the Gansu Provincial Clinical Research Center for Urological Diseases. Cisplatin (No. HY-17394, MCE) was used in this study.

### 2.3. Immunohistochemistry (IHC)

Paraffin-embedded tumor tissue specimens were obtained from 62 patients with BC who underwent surgery at the Department of Urology, the Second Hospital of Lanzhou University, between January 2020 and June 2023. Detailed clinical and pathological data are provided in Supplementary Table S2. This study was approved by the Ethics Committee of The Second Hospital of Lanzhou University (2026A-581) and adheres to the ethical principles outlined in the Declaration of Helsinki.

Human BC tissues and mouse tumor samples from mice were fixed in 10% neutral-buffered formalin, dehydrated via a graded ethanol series, embedded in paraffin, and subsequently cut into serial sections at a thickness of 4 μm. IHC detection was performed using the UltraSensitive™ SP IHC kit (KIT-9710, Maxin Biotechnology, Rockville, MD, USA) with the protocol. Staining was developed using DAB, followed by hematoxylin counterstaining, dehydration with graded ethanol, clearing with xylene, and mounting with neutral resin. IHC staining was scored using a double-blind method. Two senior pathologists independently reviewed and scored the slides, and in case of disagreement, consensus was reached through consultation. A semi-quantitative composite scoring system was employed: Staining intensity was graded as follows: 1 (negative, no staining), 2 (pale yellow, weakly positive), 3 (brownish-yellow, moderately positive), and 4 (dark brown, strongly positive). The percentage of positive cells was categorized based on the proportion of positive cells relative to the total number of tumor cells: 1 point (≤10%), 2 points (11–25%), 3 points (26–50%), 4 points (51–75%), and 5 points (>75%). The final composite score was calculated as the product of the staining intensity score and the percentage of positive cells score.

### 2.4. RNA Extraction and qRT-PCR

Total RNA was extracted using TRIzol reagent (BS258A, Biosharp, Beijing, China) and reverse transcribed into complementary DNA (cDNA) using the Evo M-MLV reverse transcription kit (AG11705, Accurate Biology, Guangzhou, China). Quantitative reverse transcription polymerase chain reaction (qRT-PCR) was performed with SYBR Green Pro Taq HS (AG11701, Accurate Biology) on a CFX96 Touch real-time PCR system (Bio-Rad, Hercules, CA, USA). Relative expression levels were calculated using the 2^−ΔΔCt^ method. The primers used were as follows:

POLD1: Forward-AGCAGGTCAAGGTCGTATCC, Reverse-AGCGTGGTTAACACAGGTTG; MAZ: Forward-TTAGGAAGTGAGCAACGGCT, Reverse-CACAGGAGAAACACCACGGA; ACTIN: Forward-GAGACCTTCAACACCACCAGC, Reverse-AGGAAGGAAGGCCTGGAAGAG.

### 2.5. Protein Extraction and Western Blotting (WB)

Total proteins were extracted using RIPA lysis buffer containing protease and phosphatase inhibitors (R0010, Solarbio, Beijing, China). Protein concentrations were determined using the BCA assay, followed by electrophoresis. The PVDF membrane containing proteins from the SDS-PAGE gel was blocked with 5% skim milk and incubated overnight with the primary antibody at 4 °C. WB was performed using the Odyssey CLX near-infrared dual-color fluorescence imaging system (Licor, Lincoln, NE, USA) and the corresponding fluorescent secondary antibodies (Licor, USA). The primary antibodies used in this study were as follows:β-Actin (AC026, Abconal, Woburn, MA, USA); POLD1 (ab186407, Abcam, Cambridge, UK); γH2AX (83307-2-RR, Proteintech, Rosemont, IL, USA); BCL2 (12789-1-AP, Proteintech); BAX (0599-2-Ig, Proteintech); Cleaved-Caspase3 (25128-1-AP, Proteintech); N-cadherin (22018-1-AP, Proteintech); E-cadherin (60186-1-Ig, Proteintech); Snail (82214-2-RR, Proteintech); Vimentin (10366-1-AP, Proteintech); ATM (27156-1-AP, Proteintech); p-ATM (AP1030, Abconal); RAD51 (14961-1-AP, Proteintech); 53BP1 (20002-1-AP, Proteintech); XRCC6 (66607-1-Ig, Proteintech); XRCC5 (16389-1-AP, Proteintech); DNA-PKcs (85370-2-RR, Proteintech); MAZ (21068-1-AP, Proteintech); Flag tag (AE092, Beyotime, Shanghai, China); HA tag (AG8057, Beyotime).

### 2.6. Lentiviral Construction and Stable Transfection of Cells

Knockdown and overexpression plasmids for POLD1, ATM, and MAZ were purchased from Miaoling Biology (Wuhan, China). Lentiviral packaging was performed by transfecting 293T cells with two packaging plasmids, psPAX2.0 and PMD2.0, along with the target plasmid (PLKO.1-EGFP was used as a positive control) using linear polyethylenimine (PEI) (40816ES02, Yasen, Shanghai, China) as the transfection reagent. Lentivirus was harvested at 48 and 72 h post-transfection. Approximately 2 × 10^5^ BC cells in the logarithmic growth phase were seeded into 6-well plates. The following day, the constructed lentivirus and the transfection enhancer Polybrene (HY-112735, MCE, Junction, NJ, USA) were added. After 72 h, cells were selected for 1 week using 500 μg/mL G418 or 2 μg/mL puromycin. The expression levels of POLD1, ATM, and MAZ were confirmed by qRT-PCR and WB.

### 2.7. Cell Viability Assay

Cell viability was assessed using the Cell Counting Kit-8 (CCK-8, catalog number: K1018, ApexBio, Houston, TX, USA). To evaluate the half-maximal inhibitory concentration (IC50), approximately 3 × 10^3^ cells per well of each cell line were seeded into a 96-well plate. After 24 h, the cells were treated with varying concentrations of the compound (0, 1, 2, 4, 8, 16, 32 μM) and cultured for an additional 48 h. Subsequently, 10 μL of CCK-8 reagent was added to each well, and the plates were incubated at 37 °C for 1 h. Absorbance was then measured at 450 nm.

### 2.8. Transwell Assay

BC cells were seeded in the upper chamber at a density of 2 × 10^4^ cells, while 700 μL of medium containing 20%FBS was added to the lower chamber. After 24 h of incubation at 37 °C, the migrated cells were fixed with 4% paraformaldehyde for 20 min, followed by staining with 0.1% crystal violet (C0121, Beyotime) for 30 min. Images were captured using an optical microscope, and the number of migrated cells was quantified using ImageJ software, version 1.8.0.

### 2.9. Wound-Healing Assay

BC cells were seeded at a density of 3 × 10^5^ cells per well in a 6-well plate. Once the cells reached 90% confluence, a sterile 200 μL pipette tip was used to create a scratch perpendicular to the bottom of the well. After gently washing away detached cells with PBS, the medium was replaced with fresh medium containing 6% FBS for continued culture. Images were captured at the same location at 0 and 24 h. The area of the residual scratch was measured using ImageJ software, and the scratch healing rate was calculated.

### 2.10. Clonogenic Assay

BC cells were seeded at a density of 500 cells per well in a 6-well plate and cultured for 10–14 days until clonogenic colonies formed. Cells were treated with varying concentrations of cisplatin (0, 2, 4, 8, 16 μM) for 48 h. After treatment, cells were fixed with 4% paraformaldehyde for 30 min, stained with 1% crystal violet for 30 min, and gently rinsed under running water. The plates were air-dried, and colonies were photographed for counting.

### 2.11. Immunofluorescence (IF) and Comet Assay

BC cells were seeded in 12-well plates. After 24 h of treatment with PBS or cisplatin, the cells were fixed with 4% paraformaldehyde at room temperature for 20 min and permeabilized with 0.1% Triton X-100 for 10 min. The cells were then blocked with 3% bovine serum albumin (BSA) at room temperature for 1 h. The corresponding primary antibody was added dropwise and incubated overnight at 4 °C. After three washes with PBS (5 min each), the cells were incubated with a fluorescently labeled secondary antibody in the dark for 1 h at room temperature. Nuclei were stained with DAPI (C1006, Beyotime) for 5 min, and the cells were mounted with an anti-fade mounting medium. Images were captured using a fluorescence microscope (Olympus, Tokyo, Japan).

For the Comet assay, BC cells treated with cisplatin for 48 h were collected, resuspended in PBS to a concentration of 2 × 10^5^ cells/mL. The Comet assay was performed using the Comet Assay Kit (C2041S, Beyotime) according to the manufacturer’s instructions. The samples were observed and photographed under a fluorescence microscope, and DNA damage was quantified using CASP software, version 1.2.2 (Comet Assay Software Project, SourceForge, San Diego, CA, USA). The percentage of tail DNA (% Tail DNA) was used as a quantitative indicator of DNA damage.

### 2.12. Cell Cycle and Apoptosis Analysis

Cell cycle detection was performed with a cell cycle analysis kit (CCS01, Multi Sciences, Hangzhou, China) following the manufacturer’s protocols, and samples were analyzed on a CytoFLEX flow cytometer (Beckman, Brea, CA, USA). FlowJo V10.0 software was used to quantify cell cycle distribution. Cell proliferation activity was further evaluated via the proliferation index (PI), calculated as PI = (S + G2/M)/(G0/G1 + S + G2/M), which represents the fraction of cells in proliferative cell-cycle phases.

BC cells were seeded in six-well plates and cultured for 48 h following treatment with PBS or cisplatin. The cells were then harvested and washed twice with PBS. Apoptosis was analyzed using the Annexin V-APC/7-AAD dual-staining kit (AP105, Multi Sciences). Cells were incubated in the dark at room temperature for 15 min, and apoptosis analysis was performed using a CytoFLEX flow cytometer (Beckman Coulter, Brea, CA, USA) with CytExpert 2.0 software. The total apoptosis rate was calculated as the sum of the early and late apoptosis rates.

### 2.13. DNA Repair Assay

The DR-GFP and EJ5-GFP reporter gene systems were used to assess the repair efficiency of HR and non-homologous end joining (NHEJ) pathways [30]. 293T cells stably expressing shPOLD1 or POLD1oe, along with their corresponding empty vectors, were seeded at a density of 5 × 10^4^ cells per well in a 6-well plate. After 24 h, the DR-GFP (or EJ5-GFP) reporter plasmid and the I-SceI endonuclease expression plasmid were co-transfected using the PEI transfection reagent. Then, 48 h post-transfection, cells were harvested, and the proportion of GFP-positive cells was determined by flow cytometry to evaluate the efficiency of double-strand break repair.

### 2.14. Co-Immunoprecipitation (Co-IP)

Co-IP experiments were performed using the Beaver Beads™ Protein A/G IP Kit (22202-20, Beaver Nano-Technologies, Suzhou, China). Total proteins were extracted from T24CisR and UMUC3CisR cells. After lysis, the supernatant was collected by centrifugation. To detect the endogenous interaction between POLD1 and ATM, the supernatant was incubated overnight at 4 °C with anti-POLD1 antibody and Protein A/G beads. Following washing with buffer, loading buffer was added, and the samples were boiled to denature and elute the proteins. The eluted proteins were analyzed by WB. For exogenous validation, 293T cells were transfected with Flag-POLD1 and/or HA-ATM plasmids. Forty-eight hours post-transfection, cells were harvested, lysed, and subjected to immunoprecipitation using anti-Flag or anti-HA antibodies. WB was performed to detect POLD1 and ATM protein expression.

### 2.15. Chromatin Immunoprecipitation–Polymerase Chain Reaction (ChIP-PCR) and Dual Luciferase Assay

ChIP-PCR was performed to identify binding sites between MAZ and the POLD1 promoter region. The assay was conducted using T24CisR and UMUC3CisR cells, following the standard protocol provided in the ChIP assay kit (RK20258, Abclonal, Woburn, MA, USA). Immunoprecipitation was carried out using an anti-MAZ antibody (21068-1-AP, Proteintech, Rosemont, IL, USA) and an IgG control antibody. After purifying the ChIP eluate, PCR was performed to assess the enrichment of specific sites within the POLD1 promoter. The primers for the three binding sites are as follows: Site1: F-AAGCAGAGGAAGAGGAAGGC, R-CTTACACCTCAAGCCCAGCA;

Site2: F-GCACATAGGGTCCCAGTGTT, R-TCCTTCAAGGGTGAGTTCTGA;

Site3: F-CGCCCGGTACACTACTCTTAG, R-GGTTGGATGGAGGGATGCAA.

To further confirm the MAZ-binding site on the POLD1 promoter, a dual-luciferase reporter assay was performed. The wild-type (WT: CCCCTCCC) and mutant (MUT: GTACACTC) sequences of the POLD1 promoter were used to construct luciferase reporter plasmids, p-MUT(site1)-Fluc-SV40-hRluc and p-WT(site1)-Fluc-SV40-hRluc. The pcDNA3.1-MAZ plasmid was used to express the transcription factor. After transfection into 293T cells, luciferase activity was measured using the Dual-Luciferase Reporter Assay Kit (11402ES, Yeasen, Shanghai, China) according to the manufacturer’s instructions. Firefly luciferase activity was normalized to Renilla luciferase activity.

### 2.16. Animal Experiments

Four-week-old female BALB/c nude mice were purchased from Lanzhou Veterinary Research Institute, Chinese Academy of Agricultural Sciences. All animal experimental procedures were performed in accordance with the Guide for the Care and Use of Laboratory Animals and approved by the Ethics Committee of the Second Hospital of Lanzhou University (D2026-503). For the subcutaneous xenograft model, genetically modified UMUC3CisR cells were resuspended in sterile PBS at a density of 5 × 10^6^ cells per 100 μL and subcutaneously injected into the right axilla of nude mice, with five mice assigned to each experimental group. Tumor formation was observed approximately 7 days after cell inoculation, followed by intraperitoneal administration of cisplatin (3 mg/kg) every 3 days. Starting from day 10 post-inoculation, tumor volume was measured every 5 days using the formula: V (mm^3^) = L × W^2^/2, where L represents the longest diameter and W denotes the shortest diameter of the tumor. For tissue harvesting, mice were first euthanized via intraperitoneal injection of pentobarbital sodium at a lethal dose of 150 mg/kg. After the disappearance of vital signs, cervical dislocation was performed as a secondary procedure to confirm irreversible death. All operations complied with standardized animal euthanasia guidelines. Afterward, the whole subcutaneous tumor mass was isolated, photographed, and weighed.

### 2.17. Statistical Analysis

All quantitative data are expressed as mean ± standard deviation (SD), with the number of independent biological replicates (n) explicitly stated in each figure legend. For two-group comparisons, statistical significance was determined using two-tailed Student’s *t*-test. For multi-group comparisons, one-way or two-way analysis of variance (ANOVA) was performed, followed by Tukey’s post hoc test for pairwise comparisons. All statistical analyses were conducted using GraphPad Prism 10.0 software. A *p* < 0.05 was considered statistically significant. * *p* < 0.05, ** *p* < 0.01, *** *p* < 0.001, **** *p* < 0.0001.

## 3. Results

### 3.1. POLD1 Is Highly Expressed in Cisplatin-Resistant BC Tissues and Cells

**Figure 1:**
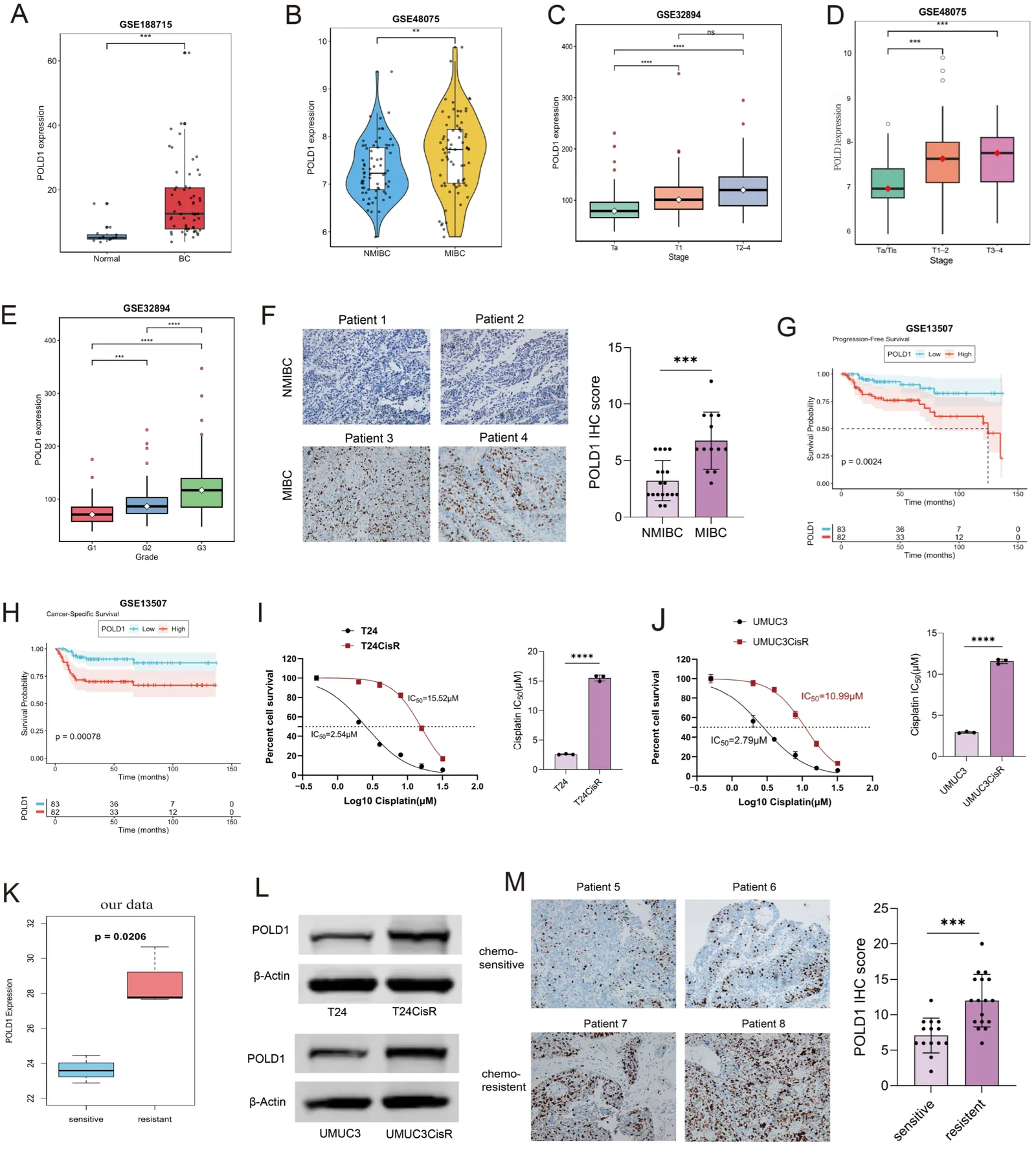
POLD1 is highly expressed in cisplatin-resistant BC tissues and cells. (**A**) Relative expression of POLD1 in BC tissues and adjacent normal tissues based on the GEO dataset GSE188715. (**B**) Comparison of POLD1 expression between NMIBC and MIBC samples in the GEO dataset GSE48075. (**C**–**E**) In the GSE32894 and GSE48075 cohorts, POLD1 expression is shown to be closely related to BC staging. (**F**) In IHC samples, POLD1 expression in NMIBC (*n* = 18) is lower than in MIBC (*n* = 13). (**G**,**H**) In the GSE13507 cohorts, POLD1 expression is associated with poorer prognosis in BC. (**I**,**J**) The CCK-8 assay was applied to determine the IC50 of cisplatin in cisplatin-sensitive and cisplatin-resistant T24 (*n* = 3, *p* < 0.0001, 95% CI: 12.20–13.75) and UMUC3 (*n* = 3, *p* < 0.0001, 95% CI: 8.18–9.13) cell lines. (**K**) Comparison of POLD1 expression between cisplatin-sensitive and cisplatin-resistant groups based on sequencing data. (**L**) WB analysis of POLD1 protein expression in cisplatin-sensitive and cisplatin-resistant T24 and UMUC3 cell lines. (**M**) IHC analysis of POLD1 expression in chemoresistant (*n* = 17) and chemosensitive (*n* = 14) clinical tissue groups. ns, not significant, ** *p* < 0.01, *** *p* < 0.001, **** *p* < 0.0001.

In the GSE188715 and GSE13507 cohorts, the mRNA level of POLD1 was significantly elevated in BC specimens relative to normal bladder tissues (Figure 1A and Figure S1A). Furthermore, data derived from the GSE13507, GSE32894 and GSE48075 cohorts revealed that POLD1 expression was correlated with tumor stage and pathological grade of BC (Figure 1B–E and Figure S1B–E). IHC staining results demonstrated that POLD1 expression was weaker in non-muscle-invasive bladder cancer (NMIBC) compared with muscle-invasive bladder cancer (MIBC), which could effectively distinguish the progression of muscular invasion (Figure 1F). Kaplan–Meier (KM) survival analysis indicated that high POLD1 expression was associated with dismal prognosis in BC patients (Figure 1G,H and Figure S1F). To validate the correlation between POLD1 and cisplatin resistance in BC cells, we constructed cisplatin-resistant cells T24CisR and UMUC3CisR and confirmed their stable drug-resistant phenotypes (Figure 1I,J). Transcriptomic sequencing of the parental and cisplatin-resistant BC cells revealed significantly upregulated POLD1 expression in the cisplatin-resistant cells (Figure 1K). WB and IHC results revealed that POLD1 expression was markedly upregulated in cisplatin-resistant BC cells and clinical tumor tissues (Figure 1L,M). Collectively, these findings suggest that POLD1 may serve as a prognostic biomarker for BC and correlate with chemoresistance.

### 3.2. POLD1 Promotes the Proliferation and Migration of Cisplatin-Resistant BC Cells

**Figure 2:**
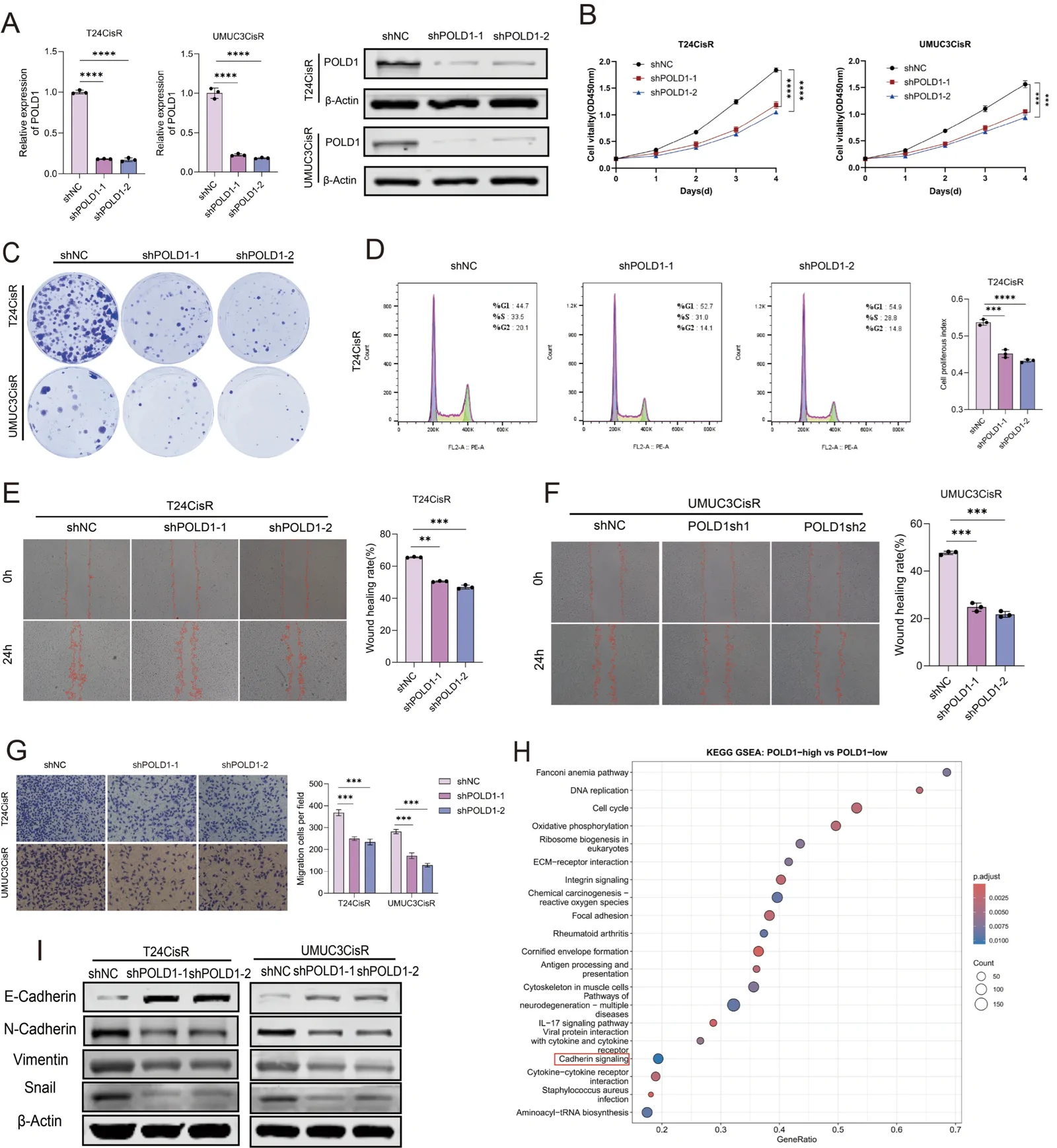
POLD1 promotes the proliferation and migration of cisplatin-resistant BC cells. (**A**) qRT-PCR and WB analyses confirm the efficiency of POLD1 knockdown in T24CisR and UMUC3CisR cells. (**B**) Knockdown of POLD1 inhibits proliferation of T24CisR and UMUC3CisR cells, (*n* = 3 independent biological replicates). (**C**) Colony formation assays evaluating clonogenic capacity of T24CisR and UMUC3CisR cells with stable POLD1 knockdown, (*n* = 3 independent biological replicates). (**D**) Flow cytometry analysis of cell cycle confirms that POLD1 knockdown suppresses T24CisR cell cycle. (**E**,**F**) Wound-healing assays demonstrate that POLD1 knockdown inhibits T24CisR and UMUC3CisR migration, (*n* = 3 independent biological replicates). (**G**) POLD1 knockdown inhibits vertical migration in T24CisR and UMUC3CisR cells, (*n* = 3 independent biological replicates). (**H**) Bubble plot of KEGG GSEA results (POLD1-high vs. POLD1-low). Each bubble represents one KEGG pathway. Bubble size indicates the count of enriched genes; color denotes the adjusted *p*-value. The x-axis shows the gene ratio of each enriched pathway. (**I**) Following POLD1 knockdown, the EMT markers N-cadherin, Vimentin and Snail are downregulated, while E-cadherin is upregulated. ** *p* < 0.01, *** *p* < 0.001, **** *p* < 0.0001.

To clarify the biological function of POLD1 in cisplatin resistance of BC, POLD1 was knocked down in cisplatin-resistant T24CisR and UMUC3CisR cells, whereas POLD1 was overexpressed in parental T24 and UMUC3 cell lines (Figure 2A and Figure S2A). Consistently, POLD1 knockdown significantly inhibited proliferation, colony formation, and invasion of T24CisR and UMUC3CisR cells (Figure 2B,C,E–G),whereas POLD1 overexpression enhanced these malignant phenotypes in T24 and UMUC3 (Figure S2B,C,E–G). Flow cytometry confirmed that POLD1 knockdown reduced cell cycle progression, whereas overexpression promoted it (Figure 2D and Figure S2D). Gene set enrichment analysis (GSEA) was performed based on the KEGG pathway database to identify differentially enriched pathways between the POLD1-high and POLD1-low expression subgroups, and the cadherin signaling pathway was confirmed to be significantly enriched in the POLD1-high group (Figure 2H). WB results demonstrated that POLD1 knockdown significantly downregulated the expression of the epithelial–mesenchymal transition (EMT)-related factors (N-cadherin, Vimentin and Snail), while upregulating the epithelial marker E-cadherin (Figure 2I). Conversely, POLD1 overexpression exerted the opposite regulatory effects on the expression of these EMT-related proteins (Figure S2H). These data suggest that POLD1 promotes the proliferation and migration of cisplatin-resistant BC cells.

### 3.3. POLD1 Contributes to Cisplatin Resistance in BC by Regulating DDR

**Figure 3:**
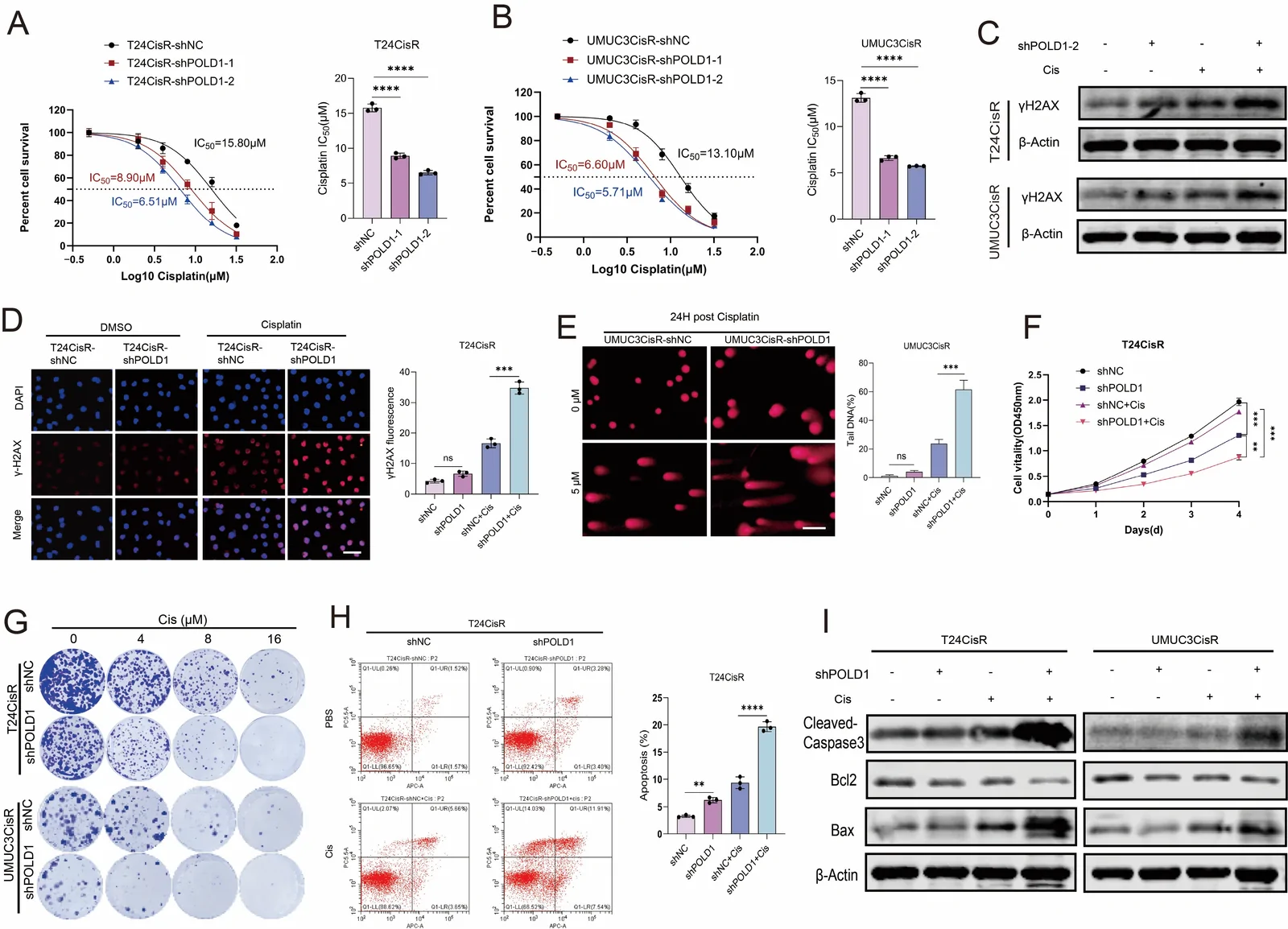
POLD1 Contributes to Cisplatin Resistance in BC by Regulating DDR. (**A**,**B**) POLD1 knockdown reduces cisplatin resistance in T24CisR (*n* = 3) and UMUC3CisR (*n* = 3) cells, as shown by lower IC_50_ values compared to NC-transfected cells (black lines). (**C**,**D**) POLD1 knockdown increases γH2AX expression and fluorescence intensity in cisplatin-treated cells. Scale bar: 50 μm. (**E**) Comet assay analysis shows longer DNA tails in POLD1 knockdown cells following cisplatin treatment. Scale bar 100 μm. (**F**) POLD1 Knockdown inhibited cisplatin-induced proliferation of T24CisR cells, (*n* = 3 independent biological replicates). (**G**) Colony formation capacity of T24CisR-shNC, T24CisR-shPOLD1, UMUC3CisR-shNC, UMUC3CisR-shPOLD1 cells exposed to cisplatin at various concentrations. (**H**) POLD1 knockdown enhances cisplatin-induced apoptosis in T24CisR cells, (*n* = 3 independent biological replicates). (**I**) WB analysis of apoptosis-related proteins following POLD1 knockdown. ns, not significant, ** *p* < 0.01, *** *p* < 0.001, **** *p* < 0.0001.

Cisplatin resistance is commonly associated with enhanced DDR capacity, increased drug efflux, inhibition of apoptosis, and remodeling of the tumor microenvironment [31]. In this study, we further explored whether POLD1 contributes to cisplatin resistance in BC through its role in DDR. Cisplatin induces DSBs in tumor DNA, which leads to an increase in γH2AX [32]. As a specific marker for DSBs, the expression level of γH2AX reflects both the extent of DSBs and the cell’s repair capacity [33]. CCK-8 cell viability assays showed that POLD1 knockdown significantly reduced the IC50 values of cisplatin-resistant BC cells and thus enhanced cellular sensitivity to cisplatin (Figure 3A,B). In contrast, POLD1 overexpression markedly increased the IC50 values of parental BC cells and attenuated cisplatin chemosensitivity (Figure S3A,B). Considering that the shPOLD1-2 exhibited a more pronounced effect on restoring cisplatin sensitivity, this sequence was selected for all subsequent functional experiments. WB and IF assays further demonstrated that, following cisplatin treatment, both the protein expression levels and IF intensity of γH2AX were markedly increased in POLD1-knockdown T24CisR and UMUC3CisR cells relative to their corresponding control groups. (Figure 3C,D and Figure S3C). Conversely, POLD1 overexpression in parental T24 and UMUC3 cells markedly reduced γH2AX fluorescence intensity (Figure S3E). These findings indicated that POLD1 depletion impaired the repair of cisplatin-induced DSBs, thereby resulting in the accumulation of DNA damage in cisplatin-resistant BC cells. Comet assays were performed to further validate these observations. The results revealed that POLD1 knockdown significantly increased the tail length and tail ratio of DNA in cisplatin-resistant cells (Figure 3E and Figure S3D), indicating an obvious impairment of cellular DDR capacity. In contrast, POLD1 overexpression substantially shortened the DNA tail length and effectively restored the DDR ability of BC cells (Figure S3G,H). CCK8 assay showed that POLD1 knockdown significantly reduced the proliferation ability of cisplatin-resistant BC cells exposed to cisplatin (Figure 3F and Figure S3H). Colony formation assays showed that POLD1 knockdown markedly reduced the colony-forming ability of cisplatin-resistant BC cells under cisplatin exposure (Figure 3G). By contrast, POLD1 overexpression significantly enhanced the colony formation capacity of parental BC cells following cisplatin exposure (Figure S3I), suggesting that POLD1 mediates cisplatin resistance by strengthening the DDR and facilitating cell survival. Flow cytometry apoptosis analysis demonstrated that POLD1 knockdown remarkably increased the apoptotic rate of cisplatin-resistant BC cells upon cisplatin stimulation (Figure 3H and Figure S3J). Consistent with these results, WB analysis (Figure 3I) further confirmed that POLD1 silencing significantly upregulated the expression of pro-apoptotic proteins BAX and cleaved caspase-3, while downregulating the level of the anti-apoptotic protein BCL2. Collectively, these results indicate that POLD1 contributes to cisplatin resistance in BC by enhancing DDR and suppressing cisplatin-induced cell apoptosis.

### 3.4. POLD1 Knockdown Suppresses Tumor Growth Under Cisplatin Chemotherapy In Vivo

**Figure 4:**
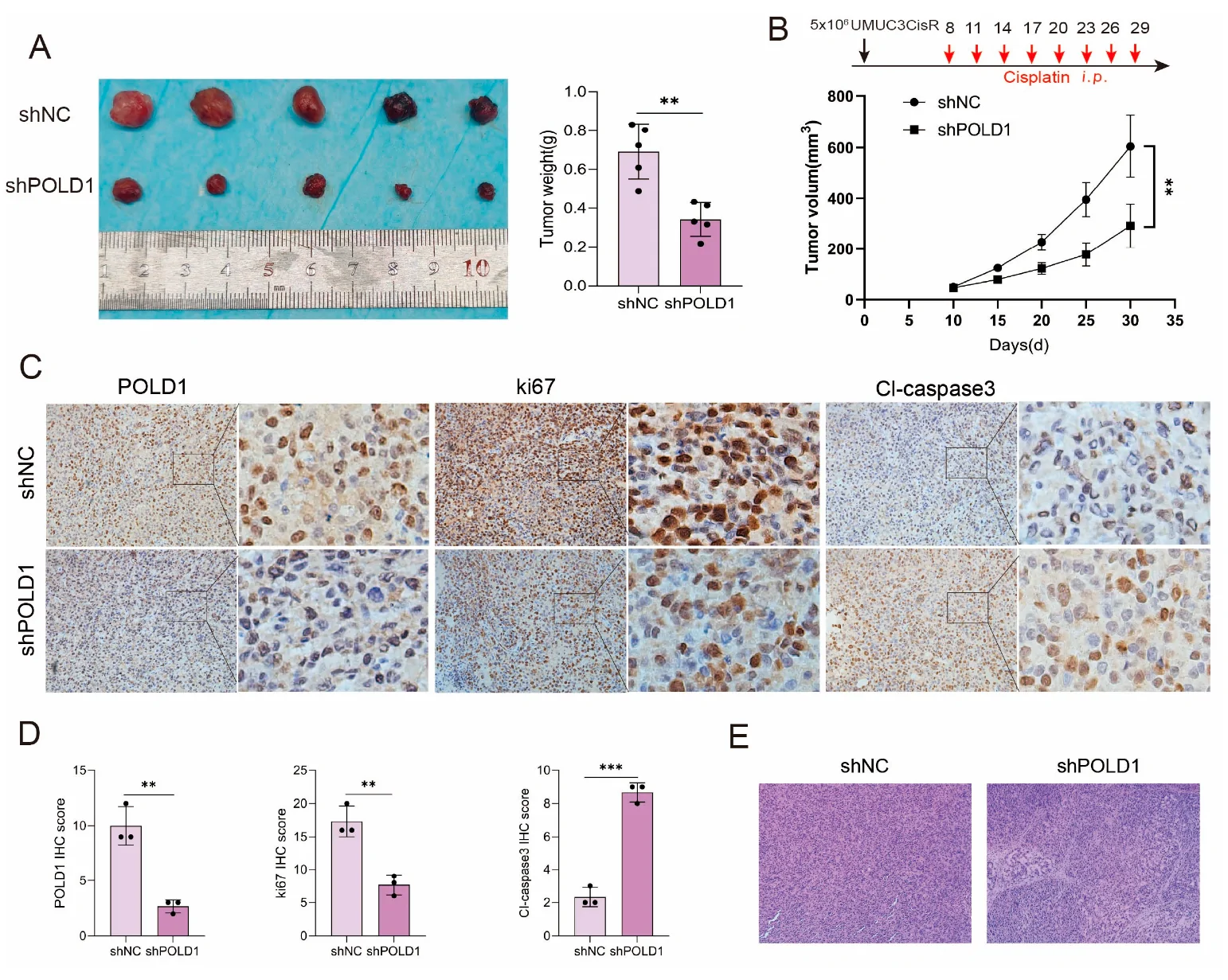
POLD1 knockdown suppresses tumor growth under cisplatin chemotherapy in vivo. (**A**,**B**) Significantly suppressed tumor growth and reduced tumor volume in POLD1 knockdown xenografts compared with control group after cisplatin administration, (*n* = 5, *p* = 0.0015, 95% CI: 155.9–466.9). Black arrows indicate subcutaneous tumor inoculation, and red arrows indicate intraperitoneal cisplatin injection. (**C**,**D**) IHC analysis revealed the expression levels of POLD1, Ki-67 and Cl-caspase3 in tumor tissues derived from POLD1 knockdown group relative to the control group. (**E**) H&E staining of tissue sections from nude mouse xenografts confirmed tumor formation. ** *p* < 0.01, *** *p* < 0.001,.

Based on in vitro findings, we used a chemotherapy model to assess whether POLD1 knockdown affects cisplatin resistance in BC cells in vivo. Consistent with the in vitro findings, in vivo animal experiments further validated that POLD1 knockdown effectively attenuates cisplatin resistance in UMUC3CisR cells. Specifically, following cisplatin treatment, both the volume and weight of xenograft tumors in the POLD1 knockdown nude mice group were significantly lower than those in the control group (Figure 4A,B). IHC staining demonstrated that the expression levels of POLD1 and Ki67 were markedly reduced, while cleaved caspase-3 expression was significantly upregulated in tumor tissues of the POLD1 knockdown group compared with the control group (Figure 4C,D). Haematoxylin and eosin (H&E) staining further verified the pathological characteristics of xenograft tumor tissues derived from nude mice (Figure 4E). These findings indicate that POLD1 knockdown suppresses BC progression and potentiates cisplatin chemosensitivity in vivo.

### 3.5. POLD1 Regulates DDR via the HR Pathway

**Figure 5:**
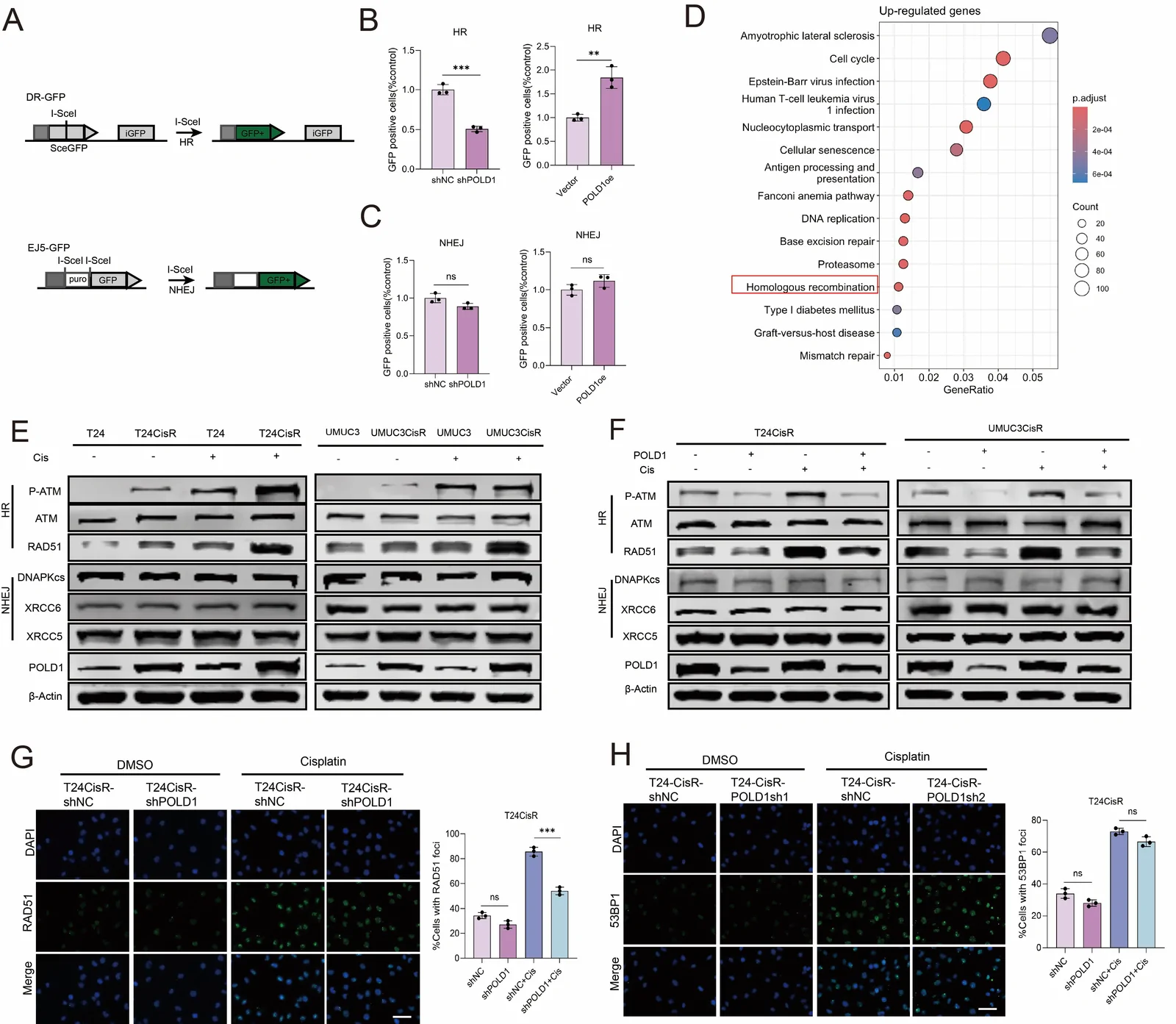
POLD1 Modulates HR Repair Efficiency. (**A**) Schematic representation of the green fluorescent protein-based HR reporter assay (DR-GFP) and the NHEJ reporter assay (EJ5-GFP). (**B**,**C**) Relative DNA repair efficiency of HR and NHEJ in T24 cells stably expressing POLD1 knockdown or POLD1 overexpression compared to their respective control groups, (*n* = 3 independent biological replicates). (**D**) KEGG enrichment analysis of genes in TCGA samples stratified by POLD1 expression levels; analysis was performed based on the KEGG database (https://www.kegg.jp/kegg/kegg1.html, accessed on 12 December 2025). (**E**,**F**) WB analysis of POLD1 and core proteins involved in HR and NHEJ quantified the relative band intensities in cisplatin-resistant cells with and without cisplatin treatment. (**G**) RAD51 (green) and DAPI (blue) staining in T24CisR cells transfected with shPOLD1, 24 h post-cisplatin treatment. Scale bar = 50 μm. (**H**) 53BP1 (green) and DAPI (blue) staining in T24CisR cells transfected with shPOLD1, 24 h post-cisplatin treatment. Scale bar = 50 μm. ns, not significant, ** *p* < 0.01, *** *p* < 0.001.

To investigate the mechanisms through which POLD1 regulates DSBs repair, we utilized the DR-GFP and EJ5-GFP reporter systems to assess HR and NHEJ repair efficiencies, respectively. The results demonstrated that POLD1 knockdown significantly impaired HR repair efficiency, while having no noticeable effect on NHEJ repair efficiency (Figure 5A–C and Figure S3K). Moreover, KEGG enrichment analysis was performed on TCGA BC samples stratified according to high and low POLD1 expression levels. The results revealed that HR-related pathways were significantly enriched in the POLD1 high-expression group (Figure 5D). WB assays demonstrated that upon cisplatin stimulation, T24CisR and UMUC3CisR cell lines exhibited substantially increased expression of RAD51 (a key HR mediator) and its upstream activated kinase p-ATM, when compared with parental BC cells. (Figure 5E). Given the high expression of POLD1 in cisplatin-resistant cells, this suggests a correlation between POLD1 and these proteins. Further analysis confirmed that POLD1 knockdown markedly reduced the expression of both RAD51 and p-ATM (Figure 5F), without affecting other key factors involved in DSBs repair. RAD51 foci formation is a hallmark of HR [34], while 53BP1 foci formation is characteristic of NHEJ repair [35]. IF analysis showed that POLD1 knockdown significantly decreased RAD51 foci formation, with no significant impact on 53BP1 foci formation (Figure 5G,H). These findings indicate that POLD1 regulates DDR through the HR pathway.

### 3.6. POLD1 Promotes ATM Phosphorylation Through Direct Interaction

**Figure 6:**
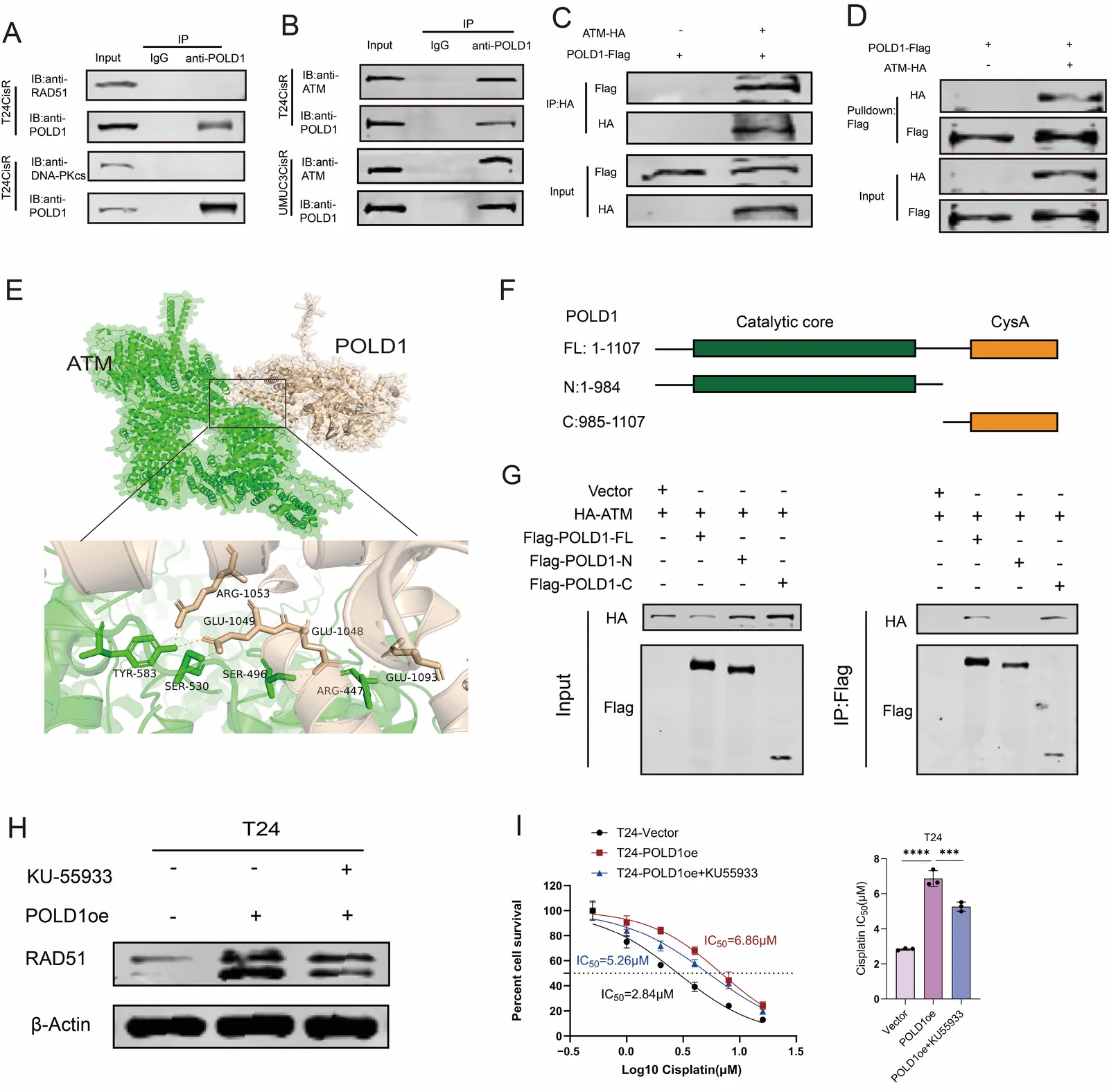
POLD1 Promotes ATM Phosphorylation Through Direct Interaction. (**A**) Co-IP assays showing no interaction between POLD1 and RAD51 or DNA-PKcs in T24CisR cells. (**B**) Co-IP assay confirming the binding of POLD1 and ATM in T24CisR and UMUC3CisR cells. (**C**) Co-IP analysis of Flag-POLD1 and HA-ATM in 293T cells. (**D**) Pull-down assay of Flag-tagged POLD1 with HA-ATM produced from 293T cells. (**E**) Predicted three-dimensional structural model of the POLD1 and ATM interaction complex from molecular docking analysis. (**F**) Schematic diagram of truncated POLD1 structural domains. (**G**) 293T cells were transfected with the above constructs. IP was performed using anti-Flag or anti-HA antibodies, followed by immunoblotting detection of cell lysates and precipitated products. (**H**) WB analysis of RAD51 and β-Actin protein levels in ATM knockdown T24 cells. (**I**) Cell viability of T24 cells with POLD1 overexpression in the presence or absence of KU-55933 was measured by CCK-8 assay. The corresponding cisplatin IC_50_ values are shown in the right panel, (*n* = 3). *** *p* < 0.001, **** *p* < 0.0001.

ATM is a critical kinase in the DSBs repair pathway and a potential therapeutic target for overcoming chemoresistance in tumors [36,37,38,39]. DSBs can induce ATM autophosphorylation at serine 1981, leading to the dissociation of ATM dimers into active monomers [35]. Activated ATM then promotes RAD51 focus formation by initiating downstream signaling, thereby facilitating HR-mediated DSBs repair. To further explore the specific mechanism through which POLD1 regulates HR-mediated DNA repair, we conducted Co-IP assays. The results showed that POLD1 exhibited no direct interaction with RAD51 or DNA-PKcs, whereas it could directly bind to ATM (Figure 6A,B). Exogenous Co-IP assays further validated the physical interaction between POLD1 and ATM. (Figure 6C), indicating that POLD1 may facilitate ATM phosphorylation through physical interaction with ATM. Furthermore, the direct interaction between POLD1 and ATM was confirmed through the pull-down assay (Figure 6D).

Protein–protein molecular docking analysis further confirmed the physical binding between POLD1 and ATM (Figure 6E). To identify the precise interaction domain responsible for POLD1-ATM binding, two truncated POLD1 mutants, including POLD1-N (1–984 aa) and POLD1-C (985–1107 aa), were constructed according to previous studies (Figure 6F). Domain mapping assays demonstrated that the ATM-binding site was located within the C-terminal structural domain of POLD1 (Figure 6G). To further verify that POLD1 confers cisplatin resistance in an ATM-dependent manner, we treated POLD1-overexpressing bladder cancer cells with the selective ATM inhibitor KU-55933 (10 μM) and performed parallel Western blotting and chemosensitivity assays. Western blot analysis showed that POLD1 overexpression markedly elevated its downstream HR effector RAD51, whereas KU-55933 treatment effectively downregulated RAD51 expression (Figure 6H). Consistently, CCK-8 viability assays demonstrated that POLD1 overexpression significantly increased the IC_50_ value of cisplatin in parental T24 cells, whereas pharmacological inhibition of ATM by KU-55933 partially reversed this chemoresistant phenotype and markedly reduced the cisplatin IC_50_ in POLD1-overexpressing cells (Figure 6I). Taken together, these findings reveal that POLD1 promotes ATM phosphorylation by physically interacting with ATM, thereby modulating HR-mediated DDR and cisplatin resistance in BC.

### 3.7. MAZ Upregulates POLD1 Transcription

**Figure 7:**
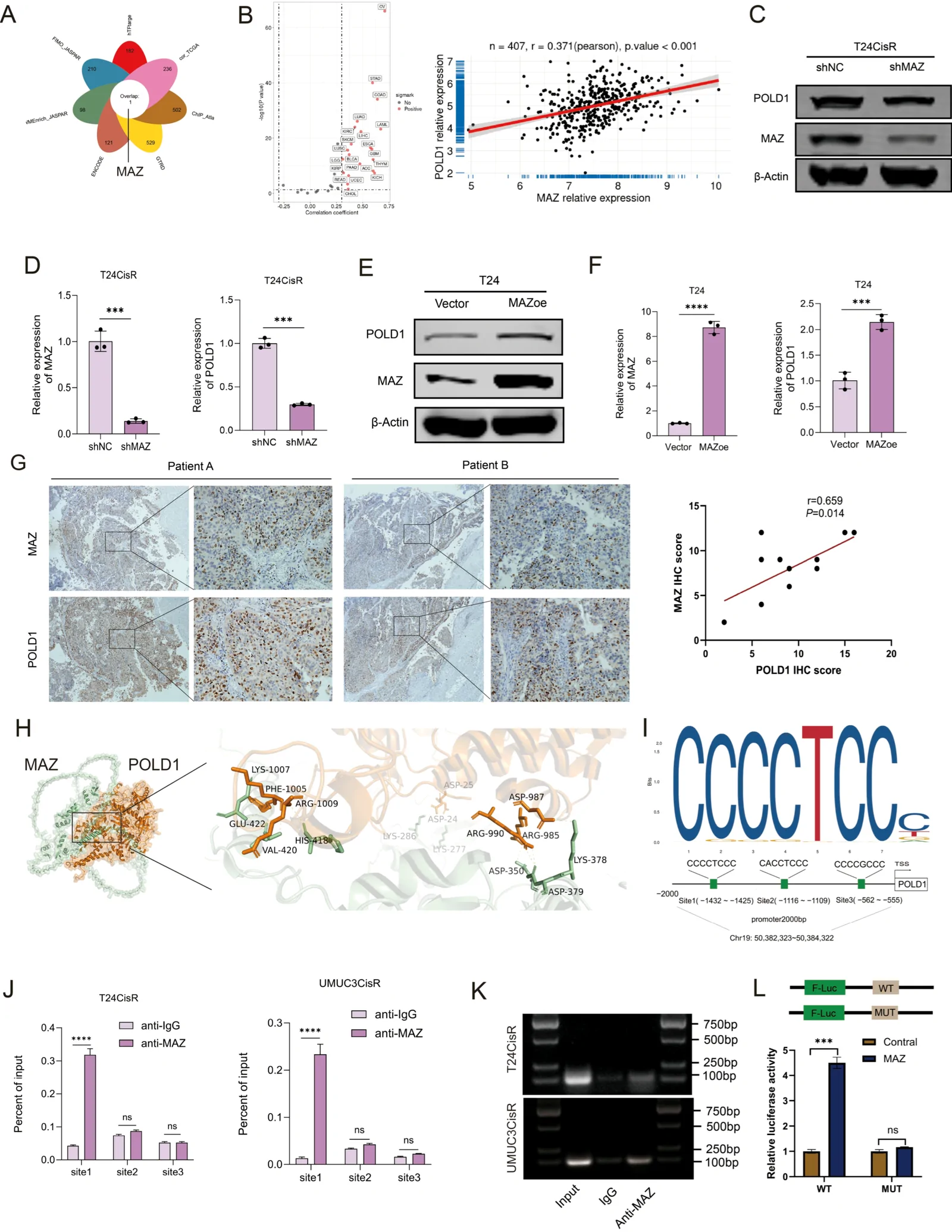
MAZ Upregulates POLD1 Transcription. (**A**) Data from the hTFtarget, ENCODE, GTRD, FIMO_JASPAR, cor_TCGA, and ChIP_Atlas databases suggest that POLD1 is a potential target gene of MAZ. (**B**) Overview of MAZ and POLD1 expression correlations across cancer types (left) and correlation analysis between MAZ and POLD1 expression in the TCGA-BLCA cohort (right). (**C**) WB analysis of POLD1 expression following MAZ knockdown in cisplatin-resistant BC cells. (**D**) qRT-PCR analysis of POLD1 expression levels in cisplatin-resistant BC cells with MAZ knockdown, (*n* = 3 independent biological replicates). (**E**) WB analysis of POLD1 expression in parental BC cells after MAZ overexpression. (**F**) qRT-PCR analysis of POLD1 expression levels in BC cells with MAZ overexpression, (*n* = 3 independent biological replicates). (**G**) IHC staining correlation analysis of MAZ and POLD1 expression in BC tissues. (**H**) Computational prediction of the potential interaction between MAZ and the POLD1 promoter region using the AlphaFold 3 model. (**I**) JASPAR database was utilized to predict and visualize the potential MAZ binding sites within the POLD1 promoter region. (**J**) ChIP-PCR analysis of MAZ occupancy at the POLD1 promoter in T24CisR and UMUC3CisR cells, (*n* = 3). (**K**) ChIP experiment using a MAZ antibody, followed by PCR amplification and agarose gel electrophoresis of the MAZ-binding site sequence from ChIP samples. The IgG antibody group served as a negative control. (**L**) Dual-luciferase reporter assay confirming the binding site 1 and regulatory relationship between MAZ and the POLD1 promoter, (*n* = 3). ns, not significant, *** *p* < 0.001, **** *p* < 0.0001.

MAZ, a transcription factor, regulates gene transcription by binding to target promoters and is involved in various physiological and pathological processes [40,41]. To investigate the upstream transcriptional regulation of POLD1, an integrated analysis of several databases (hTFtarget, KnockTF, ENCODE, ChIP-Atlas, GTRD, and ChEA) indicated that MAZ may act as an upstream regulator of POLD1 (Figure 7A). TCGA data analysis revealed a significant positive correlation between MAZ and POLD1 expression in BC tissues (Figure 7B). qRT-PCR and WB validation showed that MAZ knockdown reduced both POLD1 mRNA and protein levels, whereas MAZ overexpression increased POLD1 expression (Figure 7C–F and Figure S4A–D). IHC analysis revealed a positive correlation between POLD1 and MAZ expression (Figure 7G). Computational prediction of the potential interaction between MAZ and the POLD1 promoter region using the AlphaFold 3 model (Figure 7H). The JASPAR database predicted MAZ binding sites within the POLD1 promoter. (Figure 7I). ChIP-PCR confirmed significant enrichment at site1 (−1432 to −1426) (Figure 7J,K). To further validate the regulatory role of MAZ in POLD1 transcription, a dual-luciferase reporter assay was conducted. Luciferase assays showed that MAZ enhanced wild-type promoter activity, whereas mutation of site1 abolished this effect (Figure 7L). Overall, these findings indicate that MAZ physically interacts with the promoter region of POLD1 and activates its transcription, confirming that MAZ acts as a transcriptional activator of POLD1.

### 3.8. The MAZ-POLD1 Axis Participates in Cisplatin Resistance of BC by Regulating the DDR

**Figure 8:**
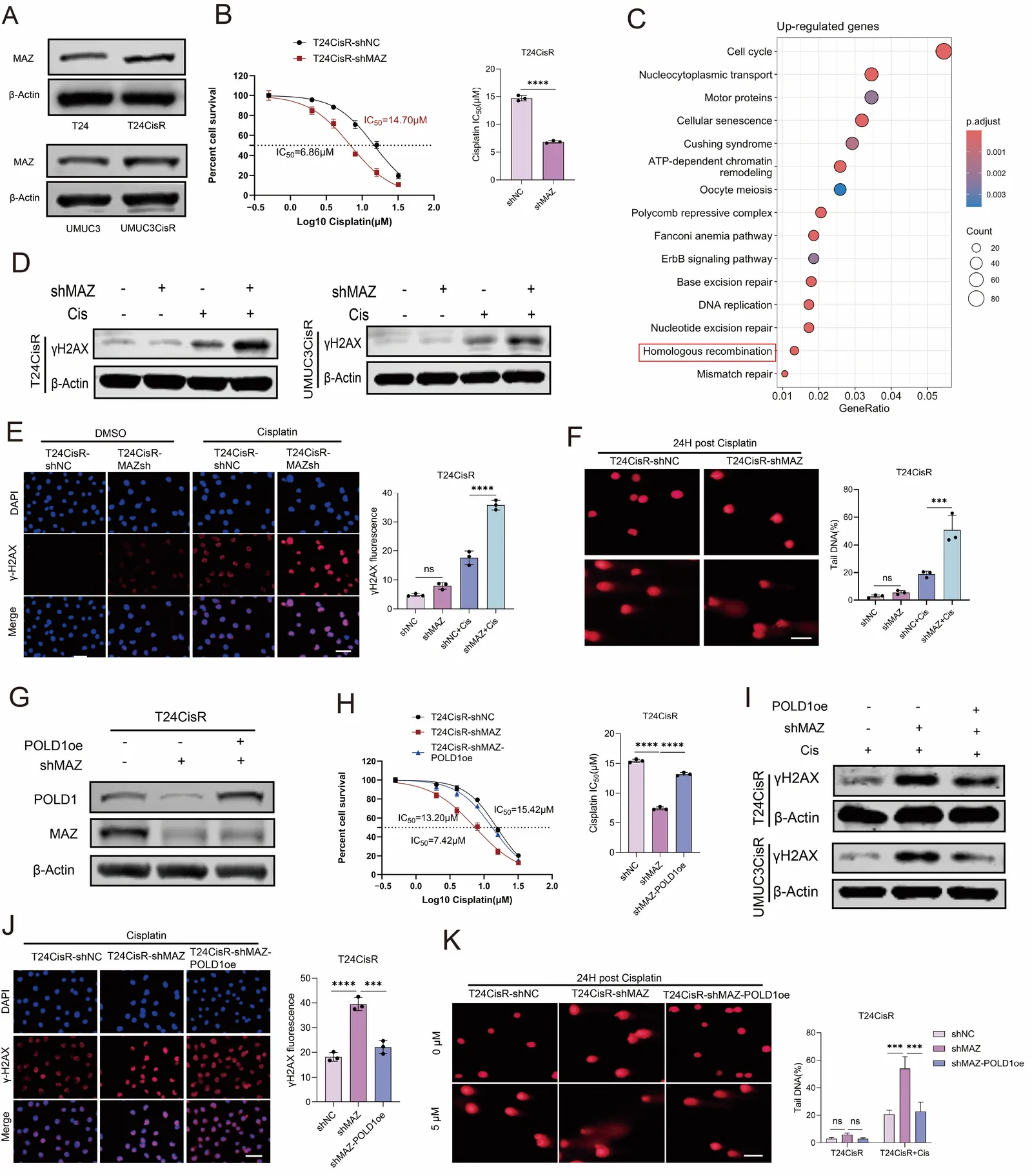
The MAZ-POLD1 axis participates in cisplatin resistance of BC via regulating the DDR pathway. (**A**) WB analysis of MAZ protein expression levels in cisplatin-sensitive and cisplatin-resistant T24 and UMUC3 cells. (**B**) MAZ knockdown reduced cisplatin resistance in T24CisR cells compared with NC-transfected cells (black line), and the corresponding IC50 values are shown in the right panel (*n* = 3). (**C**) KEGG enrichment analysis of genes in TCGA samples stratified by MAZ expression levels; analysis was performed based on the KEGG database (https://www.kegg.jp/kegg/kegg1.html, accessed on 6 March 2026).(**D**,**E**) MAZ knockdown increases γH2AX expression and fluorescence intensity in cisplatin-treated cells. Scale bar: 50 μm. (**F**) Comet assay analysis shows longer DNA tails in MAZ-knockdown cells following cisplatin treatment. Scale bar: 100 μm. (**G**) WB results of MAZ and POLD1 expression in T24CisR cells stably transfected with MAZ knockdown alone or in combination with POLD1 overexpression. (**H**) Cell viability in T24CisR cells transfected with MAZ knockdown and overexpressing POLD1, assessed by the CCK-8 assay. IC50 values are shown in the right panel, (*n* = 3). (**I**,**J**) γH2AX expression and fluorescence intensity in T24CisR cells transfected with MAZ knockdown alone or in combination with POLD1 overexpression. Scale bar: 50 μm. (**K**) Representative images of alkaline comet assays and quantitative assessment of the percentage of tail DNA in T24CisR cells transfected with NC, MAZ knockdown alone, or in combination with POLD1 overexpression, 24 h after treatment with 5 μM cisplatin. Scale bar: 100 μm. ns, not significant, *** *p* < 0.001, **** *p* < 0.0001.

WB results showed that the protein level of MAZ was significantly upregulated in T24CisR and UMUC3CisR cells compared with their parental cells (Figure 8A), which was consistent with the expression pattern of POLD1. CCK-8 assays demonstrated that MAZ knockdown markedly reduced the IC50 values of cisplatin-resistant BC cells and enhanced their sensitivity to cisplatin (Figure 8B and Figure S4E). In addition, we performed KEGG enrichment analysis on TCGA BC samples stratified by high and low MAZ expression, and the results revealed significant enrichment of pathways related to HR (Figure 8C). WB and IF assays were performed to investigate the regulatory effect of MAZ on DDR. Under cisplatin treatment, MAZ knockdown significantly increased the protein expression level and fluorescence intensity of γH2AX in T24CisR and UMUC3CisR cells compared with control groups (Figure 8D,E), suggesting that MAZ depletion impaired DSBs repair activity and led to the accumulation of DNA damage in cisplatin-resistant BC cells. Comet assays further validated these phenotypic changes. Consistent with the regulatory pattern of POLD1, MAZ knockdown markedly increased the DNA tail length and tail ratio of cisplatin-resistant cells (Figure 8F), indicating an obvious decline in cellular DDR capacity.

To further clarify the upstream and downstream regulatory relationship between MAZ and POLD1, rescue experiments were conducted in MAZ knockdown cisplatin-resistant cell lines. WB verification confirmed the successful overexpression of POLD1 in MAZ knockdown cells (Figure 8G and Figure S4F). Notably, ectopic POLD1 overexpression was incapable of rescuing reduced MAZ expression upon MAZ silencing, indicating that POLD1 could not regulate MAZ at the protein level. CCK-8 functional assays further showed that POLD1 overexpression partially reversed the enhanced cisplatin sensitivity induced by MAZ knockdown (Figure 8H and Figure S4G). WB and IF results showed that MAZ knockdown significantly increased the fluorescence intensity of γH2AX in cisplatin-resistant cells. Notably, POLD1 overexpression partially reversed this phenotype (Figure 8I,J), suggesting that POLD1 could compensate for the defective DDR function induced by MAZ depletion. Comet assays further confirmed that MAZ silencing increased the DNA tail length and tail ratio, whereas POLD1 overexpression shortened DNA tails and restored the impaired DDR capacity (Figure 8K). In summary, these results demonstrate that MAZ acts as an upstream regulator, and the MAZ-POLD1 axis modulates cisplatin resistance by regulating the DDR pathway in BC.

## 4. Discussion

Although cisplatin-based chemotherapy serves as a first-line therapeutic strategy for BC, the majority of patients eventually develop acquired chemoresistance following repeated treatment cycles, drastically compromising therapeutic responses and clinical prognosis [42,43]. Cisplatin exerts anti-tumor effects by triggering DNA damage, while tumor cells counteract cisplatin cytotoxicity through amplified DDR machinery, consequently leading to the emergence of chemoresistance [44]. Accordingly, dissecting the regulatory network of DDR and its functional mechanisms underlying chemoresistance carries crucial clinical implications for reversing cisplatin resistance and optimizing treatment outcomes of BC patients. In this study, we systematically delineated the biological function and molecular mechanism by which the MAZ-POLD1 axis modulates cisplatin resistance in BC, and validated that POLD1 mediates cisplatin resistance of BC cells via governing the HR pathway.

As the core catalytic subunit of DNA polymerase δ, POLD1 is primarily responsible for genomic DNA replication and maintenance of DDR homeostasis [45,46]. Accumulating evidence has documented aberrantly elevated POLD1 expression in a broad spectrum of malignancies, and its expression level is tightly correlated with tumor aggressiveness and patient clinical prognosis [47,48]. In BC, prior work has validated that high POLD1 expression correlates strongly with malignant tumor progression [18]. Nevertheless, the functional role and upstream regulatory mechanisms of POLD1 in cisplatin resistance of BC remain poorly characterized. In this study, we analyzed multiple public transcriptomic cohorts and verified that POLD1 expression is markedly upregulated in BC tissues; its abundance positively correlates with pathological grade, clinical tumor stage and muscular invasion, and high POLD1 expression predicts poor survival outcomes, supporting its utility as a novel prognostic biomarker for evaluating BC progression. More importantly, our results demonstrated robust upregulation of POLD1 in cisplatin-resistant cells and chemoresistant tumor tissues, establishing a solid cellular and clinical correlation between elevated POLD1 and cisplatin resistance in BC.

Functional experiments further validated the oncogenic role of POLD1 in cisplatin-resistant BC. At the cellular level, POLD1 knockdown markedly suppressed the proliferation, colony formation, and migration capacities of cisplatin-resistant BC cells, whereas POLD1 overexpression exerted the opposite effects. Epithelial–mesenchymal transition (EMT) is a pivotal biological process that modulates tumor metastasis and chemotherapy resistance. Our results demonstrated that POLD1 positively regulates EMT progression, as evidenced by the upregulation of mesenchymal markers (N-cadherin, Vimentin, and Snail) and the downregulation of the epithelial marker E-cadherin. These findings indicate that POLD1-driven malignant progression of BC is tightly linked to cisplatin resistance and EMT reprogramming, which reasonably explains the clinical phenomenon that cisplatin-resistant BC exhibits enhanced invasive and metastatic potential, as well as poor patient prognosis.

Cisplatin exerts cytotoxic effects predominantly by triggering DSBs, and aberrantly augmented DDR capacity represents a core driver of acquired cisplatin resistance in tumors. As a specific molecular biomarker of DSBs, γH2AX abundance directly mirrors the magnitude of defective cellular DDR [49]. Excessive activation of the DDR cascade efficiently counteracts chemotherapy-elicited DNA lesions, thereby blunting the therapeutic efficacy of platinum agents [50,51]. Our data revealed that POLD1 knockdown markedly attenuated cisplatin resistance. Upon cisplatin exposure, POLD1 knockdown also dramatically elevated γH2AX levels and extended comet tail length in cisplatin-resistant cells, indicating severe impairment of DDR capacity. Conversely, POLD1 overexpression produced opposing phenotypes, implying that POLD1 facilitates cisplatin resistance through potentiating DDR signalling. Furthermore, POLD1 silencing suppressed colony formation and drastically augmented apoptotic rates in cisplatin-resistant cells treated with cisplatin. Consistently with in vitro observations, in vivo xenograft assays demonstrated that POLD1 ablation robustly reversed cisplatin resistance and retarded tumor growth in nude mouse subcutaneous grafts. Collectively, POLD1 alleviates cisplatin-provoked DNA damage and blocks DNA damage-initiated apoptotic signalling by reinforcing cellular DDR competence, ultimately conferring cisplatin chemoresistance to BC cells.

Previous studies have demonstrated that DSBs repair is primarily mediated by two core pathways: HR and non-homologous end joining (NHEJ) [52]. Using the DR-GFP/EJ5-GFP reporter system, we found that POLD1 specifically modulates the efficiency of HR repair but exerts no remarkable effect on NHEJ repair. Consistently, IF staining showed that POLD1 knockdown significantly impaired the formation of RAD51 foci without altering 53BP1 foci assembly. Moreover, KEGG enrichment analysis based on TCGA BC datasets revealed significant enrichment of HR signaling in tumors with high POLD1 expression. Cumulatively, this multi-dimensional evidence demonstrates that POLD1 mediates DDR by specifically regulating the HR pathway.

As a central upstream regulator of the DSBs repair cascade, ATM orchestrates DDR and cellular apoptosis by activating downstream signaling pathways [53,54]. Upon activation, ATM further recruits recombination repair proteins such as RAD51, thereby facilitating efficient HR repair [55,56]. In this study, significantly elevated expression levels of p-ATM and RAD51 were observed in cisplatin-resistant BC cells, and their abundances were positively correlated with POLD1 expression. Mechanistically, POLD1 knockdown specifically downregulated the protein levels of p-ATM and RAD51. Co-IP assays verified a physical interaction between POLD1 and ATM. Furthermore, truncation mutation analysis mapped the binding region to the C-terminal domain (985–1107 aa) of POLD1, and protein molecular docking simulations further supported this intermolecular binding. Notably, our findings reveal a novel regulatory mechanism of POLD1. Traditionally, POLD1 is recognized primarily for its polymerase activity, which participates in DNA replication and the synthetic phase of mismatch repair. Herein, we identified a non-canonical function of POLD1 in the early stage of HR repair: POLD1 acts as a critical modulator of ATM signaling, physically interacts with ATM to promote its phosphorylation and activation, and consequently enhancing HR repair efficiency. This newly identified function broadens the current understanding of POLD1-mediated DDR regulation and provides a promising therapeutic target for reversing chemotherapy resistance. Collectively, the present study systematically elaborates the molecular mechanism by which POLD1 modulates DDR and establishes a functional POLD1-ATM/RAD51 regulatory axis in BC.

MAZ is a GC-rich transcription factor that has been predominantly reported to mediate the transcriptional regulation of proto-oncogenes, including c-MYC and Ras. As a crucial regulatory factor extensively involved in tumorigenesis and tumor progression, MAZ modulates the proliferation, invasion, metastasis, and therapeutic resistance of tumor cells via transcriptionally activating downstream target genes [57,58,59,60]. Previous studies have demonstrated that cellular experiments confirm the MAZ/NEIL3 axis suppresses DNA damage and facilitates cisplatin resistance in cancer cells [28,61]. In this study, integrated analysis of multiple public databases identified MAZ as a potential upstream regulator of POLD1. ChIP-PCR and dual-luciferase reporter assays further validated that MAZ physically interacts with to the POLD1 promoter and activates its transcriptional activity. Consistent upregulation of MAZ and POLD1 was observed in cisplatin-resistant BC cells, and clinical specimen analysis confirmed a significant positive correlation between MAZ and POLD1 expression in BC tissues. Mechanistically, MAZ knockdown downregulated POLD1 expression and recapitulated the phenotypes of increased cisplatin sensitivity and impaired HR repair induced by POLD1 deficiency. KEGG enrichment analysis revealed that BC samples with high MAZ expression exhibited prominent enrichment of the HR pathway, further supporting the synergistic role of the MAZ-POLD1 axis in HR repair. Most importantly, rescue experiments demonstrated that exogenous POLD1 overexpression could partially reverse MAZ knockdown-mediated DDR defects and cisplatin hypersensitivity in vitro. Taken together, these findings not only expand the biological function of MAZ in tumor chemoresistance but also systematically uncover the key upstream transcriptional regulatory mechanism of POLD1, providing a novel mechanistic insight into the molecular network underlying cisplatin resistance in BC.

The present study has several inherent limitations. First, cisplatin resistance arises from multifaceted and complex regulatory networks, and other well-established mechanisms, including enhanced drug efflux, intracellular drug inactivation, dysregulated apoptotic signaling, and altered autophagic activity, may also contribute to chemoresistance in BC. Second, it should be noted that our in vivo xenograft experiment only included cisplatin-treated groups and did not include vehicle-treated control cohorts, so it cannot formally establish a POLD1-by-treatment interaction effect. Formal validation of in vivo chemosensitization would require a more comprehensive in vivo experimental design that includes vehicle-treated control groups in future studies. Third, specific pharmacological inhibitors targeting POLD1 are currently unavailable, which restricts the pharmacological validation and translational application of POLD1-targeted strategies for cisplatin resistance reversal. Future studies are warranted to develop synthetic lethal approaches and effective therapeutic interventions against POLD1. Finally, our conclusions are primarily validated based on in vitro cell lines and subcutaneous xenograft models. Further verification using large-scale clinical cohorts and patient-derived xenograft models is required to comprehensively evaluate the translational potential of the MAZ-POLD1 axis as a clinical biomarker and therapeutic target for cisplatin-resistant BC.

## 5. Conclusions

The study uncovered a core regulatory role of the MAZ-POLD1 axis in modulating cisplatin resistance in BC. As a pivotal molecular node linking the upstream transcription factor MAZ to the downstream HR repair machinery, POLD1 directly interacts with ATM to facilitate ATM phosphorylation, thereby potentiating HR repair capacity and ultimately driving cisplatin resistance. Targeting the MAZ-POLD1 axis is a promising strategy to reverse cisplatin resistance and boost chemotherapy efficacy in BC.

## Data Availability

The RNA-seq data generated in this study will be deposited in a public repository before publication. Other data relevant to the study are included in the article or Supplementary Materials.

## Supplementary Materials

The following supporting information can be downloaded at: https://www.mdpi.com/article/10.3390/cancers18172892/s1. Figure S1: POLD1 was highly expressed in BC cisplatin-resistant tissues and cells; Figure S2: POLD1 promotes the proliferation and migration of BC cisplatin-resistant cells; Figure S3: POLD1 participates in BC cisplatin resistance by regulating DDR; Figure S4: The MAZ-POLD1 axis modulates cisplatin resistance by regulating DDR. Table S1: the FPKM-normalized expression matrix; Table S2: POLD1 and MAZ immunohistochemical study results and the corresponding clinical information.

## Abbreviations

The following abbreviations are used in this manuscript:

BC	Bladder cancer
DDR	DNA damage repair
DSBs	DNA double-strand breaks
GEO	Gene Expression Omnibus
HR	Homologous recombination
NHEJ	Non-homologous end joining
qRT-PCR	Quantitative real-time polymerase chain reaction
WB	Western blot
IC50	Half-maximal-inhibitory concentration
IF	Immunofluorescence
Co-IP	Co-immunoprecipitation
ChIP	Chromatin immunoprecipitation
TF	Transcription factor
