# Supplementary material for: The MAZ-POLD1 Signaling Axis Drives Cisplatin Resistance in Bladder Cancer by Activating DNA Damage Repair
Source: Cancers (Basel). 2026 Sep 7;18(17):2892. doi: 10.3390/cancers18172892 (PMC13564842; doi:10.3390/cancers18172892)
Supplement: Supplementary file 1 [file cancers-18-02892-s001.zip › Figure S1 POLD1 was highly expressed in BC cisplatin-resistant tissues and cells..pdf]

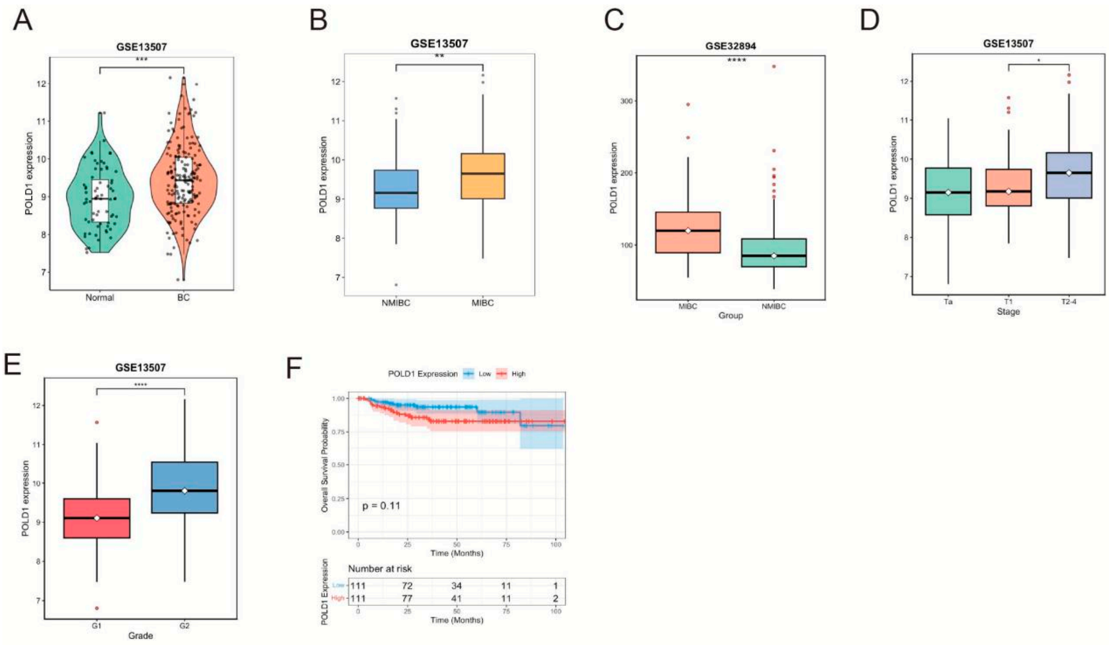

Figure S1: POLD1 was highly expressed in BC cisplatin-resistant tissues and cells. (A)Relative expression of POLD1 in bladder cancer (BC) tissues and adjacent normal tissues based on the GEO dataset GSE13507.(B-C)Comparison of POLD1 expression between non–muscle-invasive bladder cancer(NMIBC) and muscle-invasive bladder cancer (MIBC) samples in the GEO dataset GSE13507 and GSE32894.(D-E)In the GSE13507 cohorts, POLD1 expression is shown to be closely related to BLCA staging.(F)In the GSE13507 cohorts, POLD1 expression is associated with poorer prognosis in BC.
