# Supplementary material for: The MAZ-POLD1 Signaling Axis Drives Cisplatin Resistance in Bladder Cancer by Activating DNA Damage Repair
Source: Cancers (Basel). 2026 Sep 7;18(17):2892. doi: 10.3390/cancers18172892 (PMC13564842; doi:10.3390/cancers18172892)
Supplement: Supplementary file 1 [file cancers-18-02892-s001.zip › Figure S2 POLD1 promotes the proliferation and migration of BC cisplatin-resistant cells.pdf]

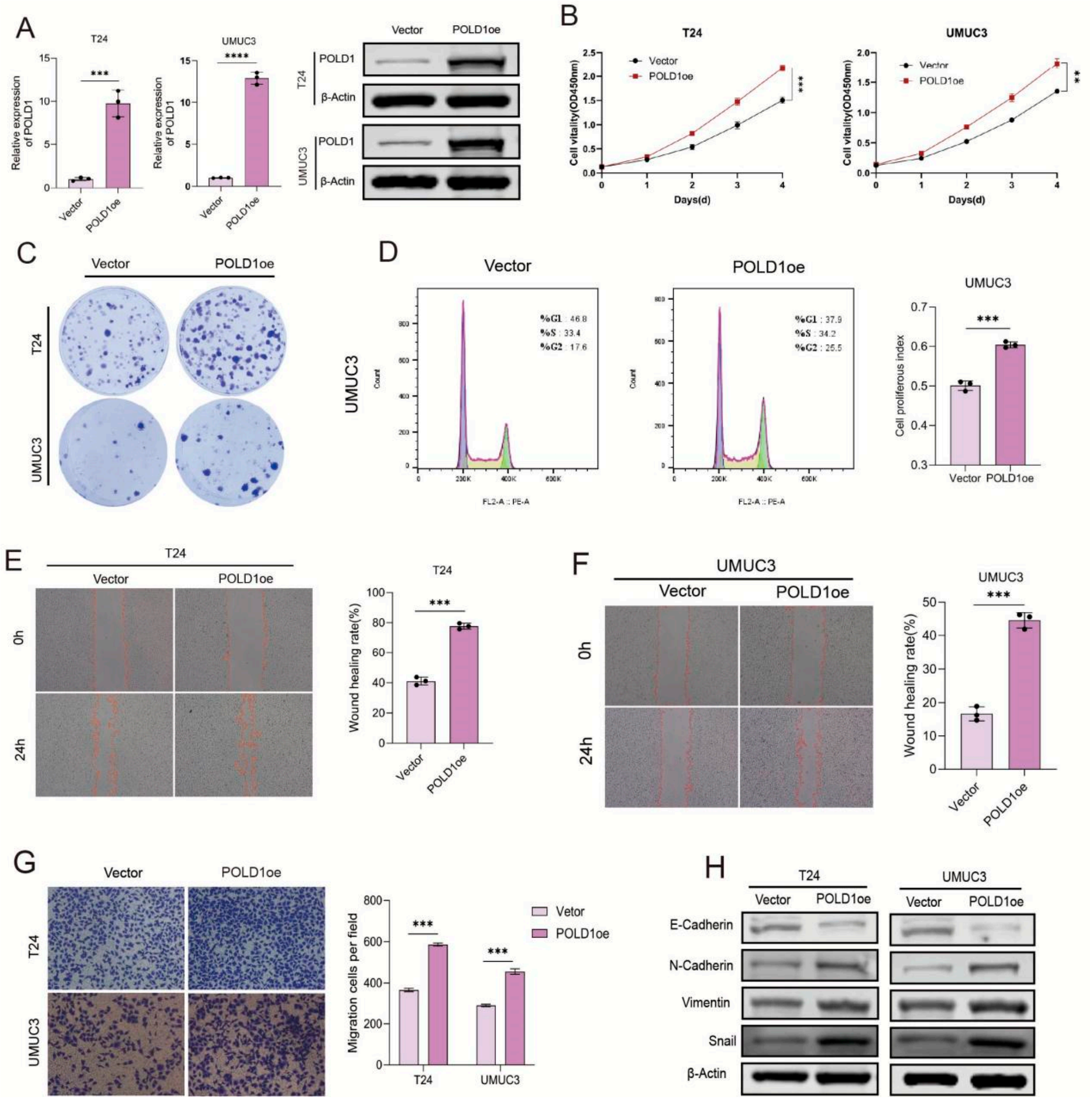

Figure S2 POLD1 promotes the proliferation and migration of BC cisplatin-resistant cells

(A) qRT-PCR and Western blot analyses confirm the efficiency of overexpression POLD1 in T24 and UMUC3 cells.(B)Overexpression POLD1 inhibits proliferation of T24 and UMUC3 cells. (C)Colony formation assays evaluating clonogenic capacity of T24CisR and UMUC3CisR cells with stable POLD1 overexpression.(D)Flow cytometry analysis of cell cycle confirms that POLD1 overexpression promotes UMUC3 cell cycle. (E-F)Cell migration in T24 and UMUC3 overexpression POLD1 cells as detected by wound healing assay.(G) POLD1 overexpression enhances migration in T24 cells.(H) In contrast, POLD1 overexpression upregulates N-cadherin and Snail, while downregulating E-cadherin.
