# Supplementary material for: The MAZ-POLD1 Signaling Axis Drives Cisplatin Resistance in Bladder Cancer by Activating DNA Damage Repair
Source: Cancers (Basel). 2026 Sep 7;18(17):2892. doi: 10.3390/cancers18172892 (PMC13564842; doi:10.3390/cancers18172892)
Supplement: Supplementary file 1 [file cancers-18-02892-s001.zip › Figure S3 POLD1 participates in BC cisplatin resistance by regulating DDR.pdf]

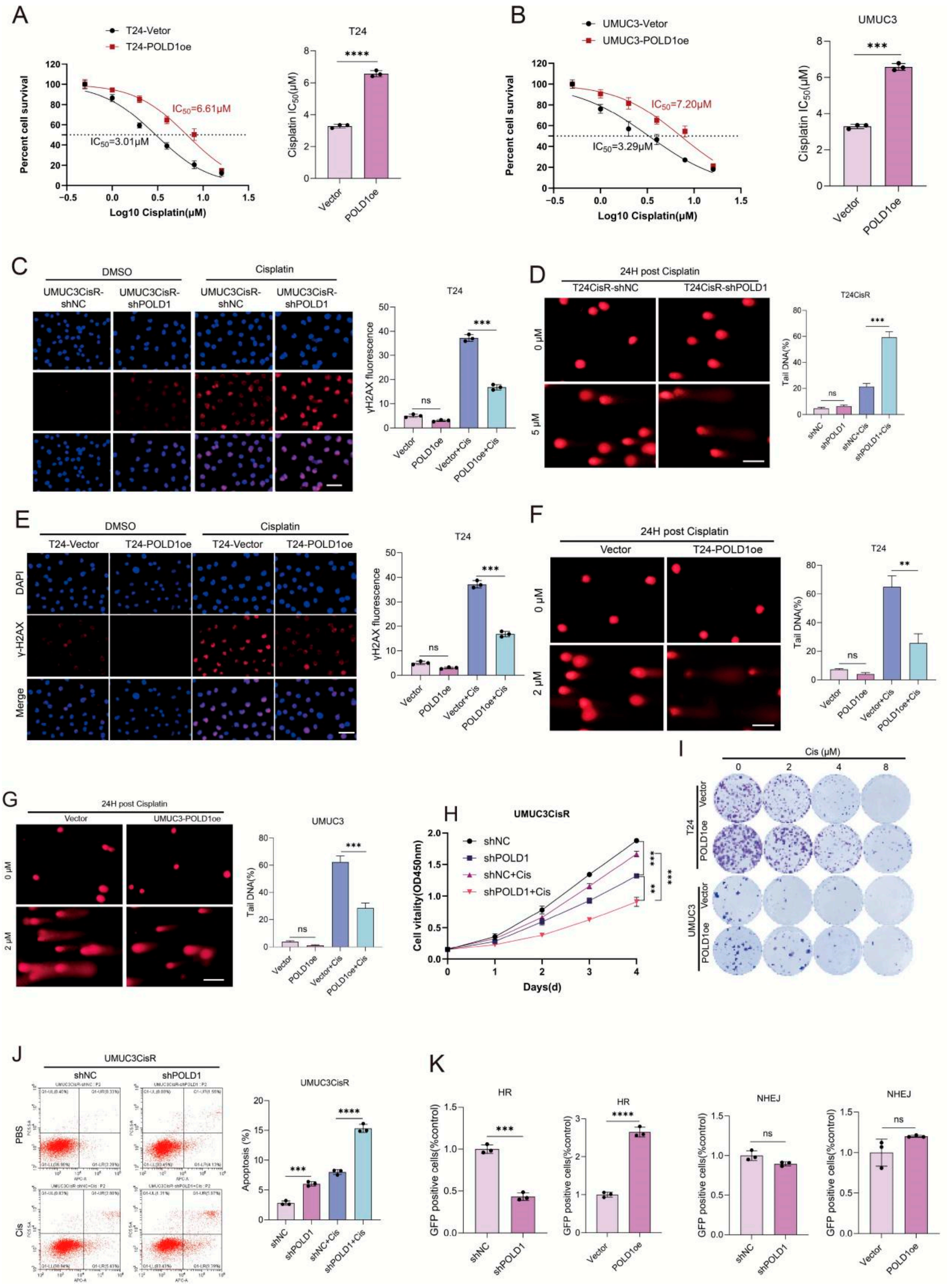

Figure S3 POLD1 participates in BC cisplatin resistance by regulating DDR

(A-B)Overexpression POLD1 enhanced cisplatin resistance in T24 and UMUC3 cells compared with control groups (black lines), and corresponding IC_{50} values are presented in the right panel. (C)Upon cisplatin treatment, POLD1 knockdown elevated γH2AX fluorescent intensity in UMUC3CisR cells.Scale bar 50 μm.(D)Comet assays revealed longer DNA tails in POLD1-knockdown cells upon cisplatin exposure.Scale bar 100 μm.(E)Upon cisplatin treatment,

POLD1 overexpression reduced γH2AX immunofluorescence intensity in T24 cells.Scale bar 50 μm. (F-G)Comet assays showed shortened DNA tails in POLD1-overexpressing cells after cisplatin exposure.Scale bar 100 μm. (H)POLD1 Knockdown inhibited cisplatin-induced proliferation of UMUC3CisR cells. (I)Colony formation assays exhibited the proliferative capacity of T24-Vector, T24-POLD1oe, UMUC3-Vector and UMUC3-POLD1oe cells treated with serially diluted cisplatin at gradient concentrations.(J)POLD1 knockdown augmented cisplatin-induced apoptosis in UMUC3CisR cells.(K)Relative DNA repair efficiency of HR and NHEJ in 293T cells stably expressing POLD1 knockdown or POLD1 overexpression compared to their respective control groups.
