# Supplementary material for: The MAZ-POLD1 Signaling Axis Drives Cisplatin Resistance in Bladder Cancer by Activating DNA Damage Repair
Source: Cancers (Basel). 2026 Sep 7;18(17):2892. doi: 10.3390/cancers18172892 (PMC13564842; doi:10.3390/cancers18172892)
Supplement: Supplementary file 1 [file cancers-18-02892-s001.zip › Figure S4 The MAZ-POLD1 axis modulates cisplatin resistance by regulating DDR..pdf]

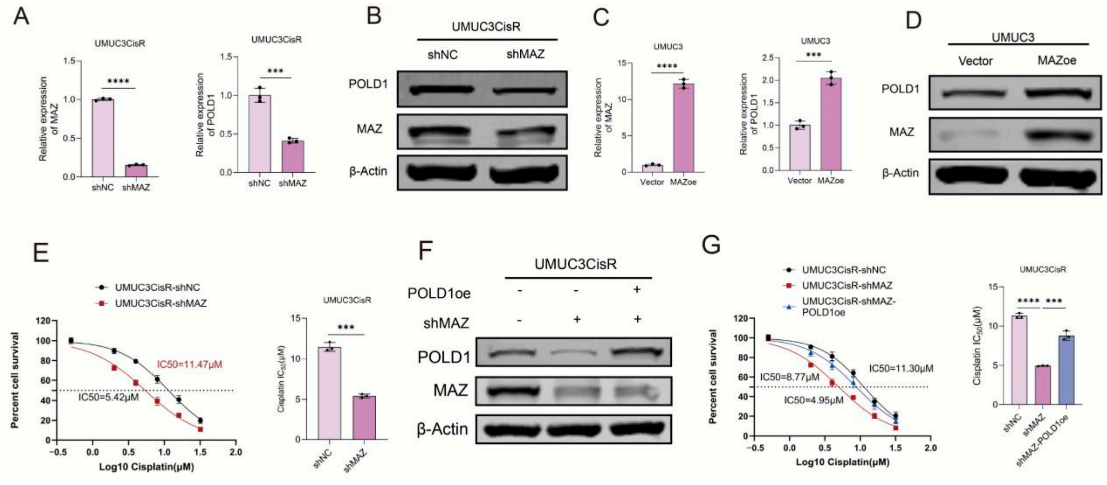

Figure S4 The MAZ-POLD1 axis modulates cisplatin resistance by regulating DDR.

(A)qRT-PCR analysis was performed to detect the mRNA expression levels of MAZ and POLD1 in MAZ-knockdown cisplatin-resistant UMUC3CisR cells.(B)WB analysis was used to examine changes in POLD1 expression following MAZ knockdown in UMUC3CisR cells.(C)qRT-PCR assays were performed to detect the mRNA expression levels of MAZ and POLD1 in MAZ-overexpressing cisplatin-sensitive UMUC3 cells.(D)WB analysis was performed to assess alterations in POLD1 expression upon MAZ overexpression in UMUC3 cells.(E)Compared with NC-transfected cells (black lines), MAZ knockdown reduced cisplatin resistance in UMUC3CisR cells, with corresponding IC_{50} values shown in the right panel.(F)WB analysis of MAZ and POLD1 expression in UMUC3CisR cells with stable shMAZ transfection alone or combined with POLD1 overexpression.(G)Cell viability in UMUC3CisR cells transfected with shMAZ and overexpressing POLD1, assessed by the CCK-8 assay. IC_{50} values are shown in the right panel.
