# Supplementary material for: The MAZ-POLD1 Signaling Axis Drives Cisplatin Resistance in Bladder Cancer by Activating DNA Damage Repair
Source: Cancers (Basel). 2026 Sep 7;18(17):2892. doi: 10.3390/cancers18172892 (PMC13564842; doi:10.3390/cancers18172892)
Supplement: Supplementary file 1 [file cancers-18-02892-s001.zip › Original WB images.pdf]

Figure1L

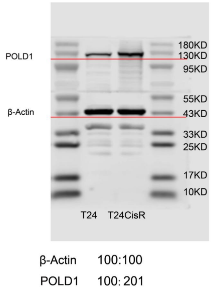

Figure2A

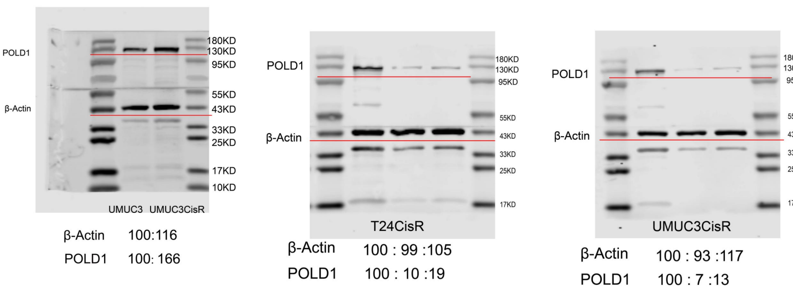

Fig.2I

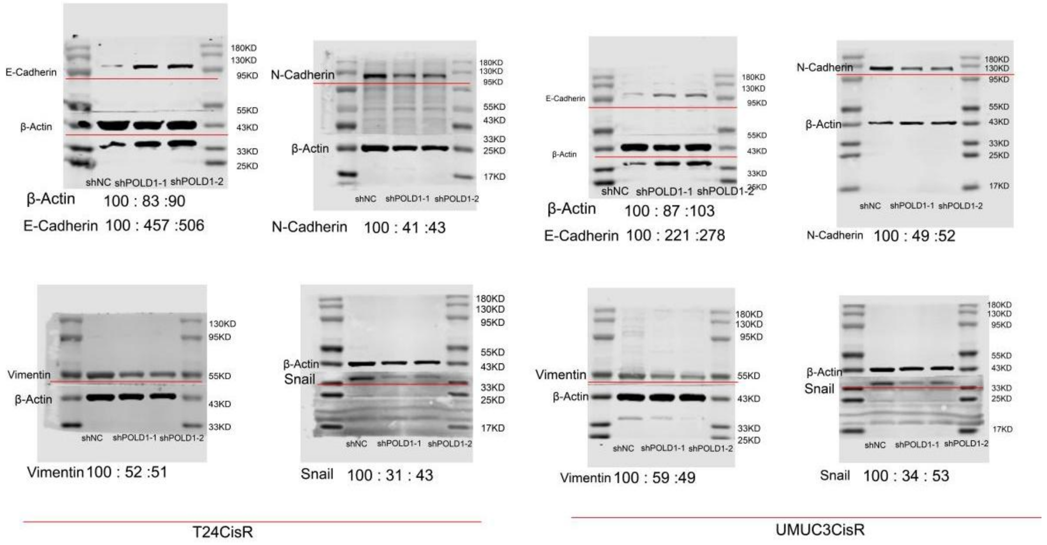

Figure3 C

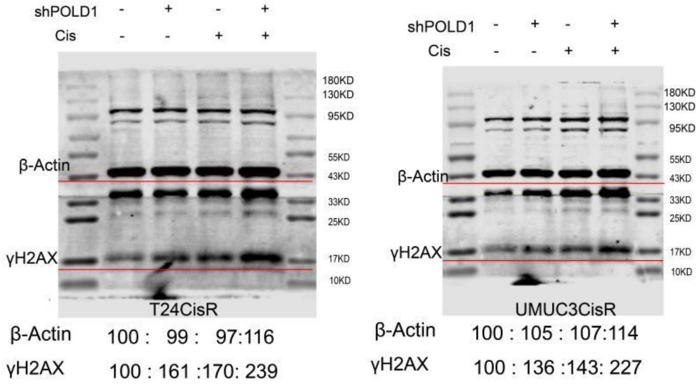

Figure3 I

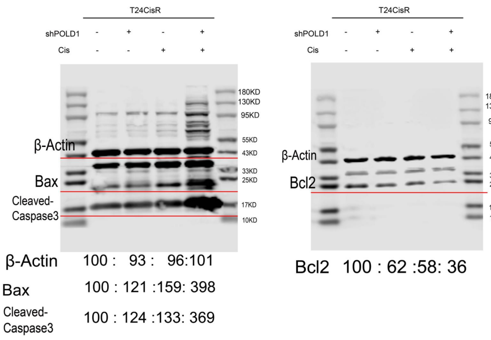

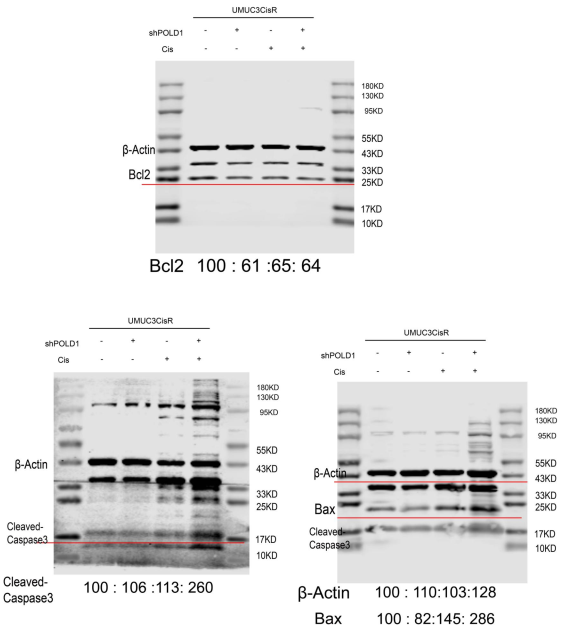

Figure5 E

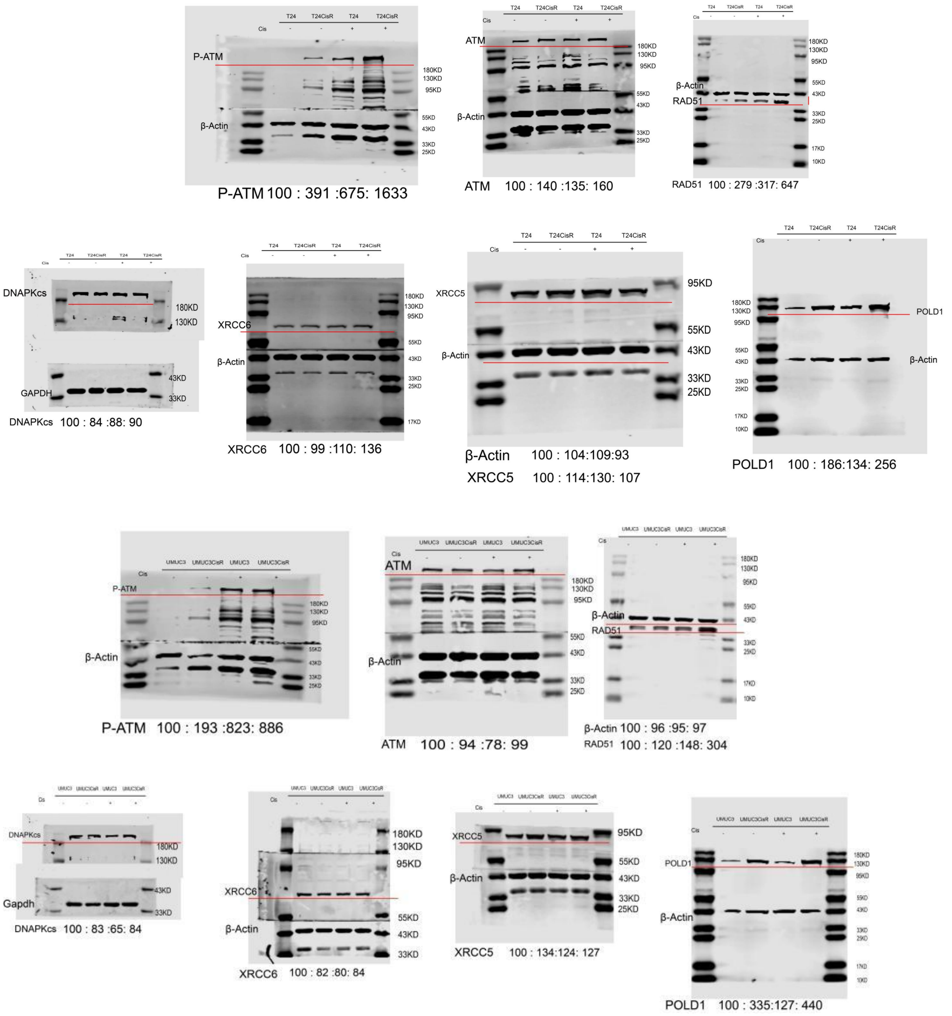

Figure5 F

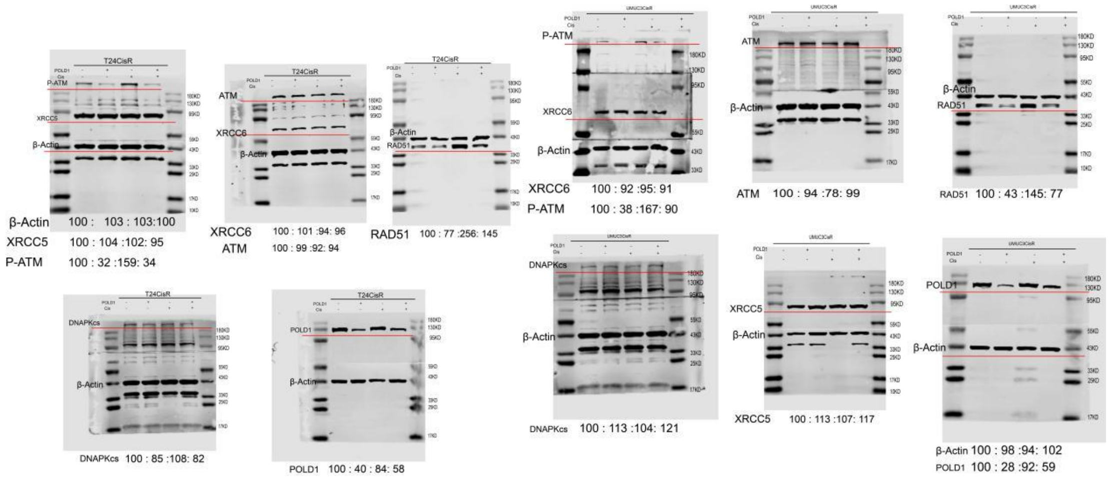

Figure6 A

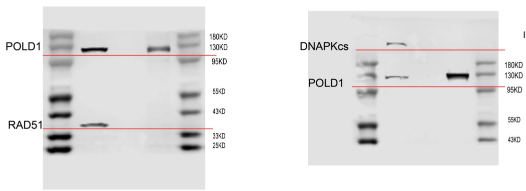

Figure6B

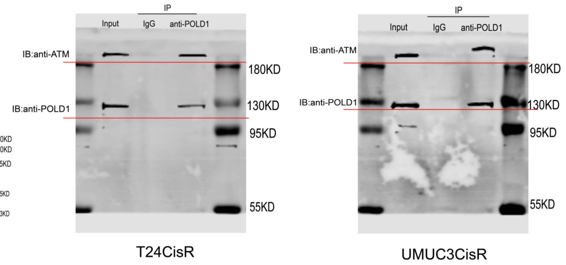

Figure6 C

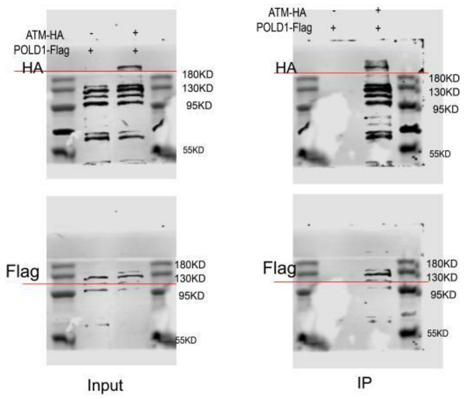

Figure6D

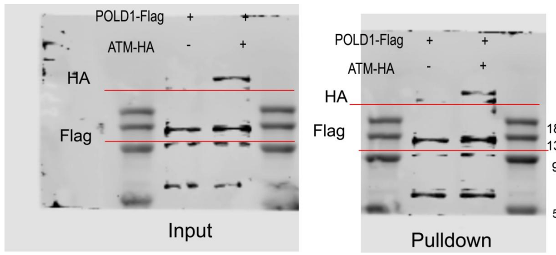

Fig6 G

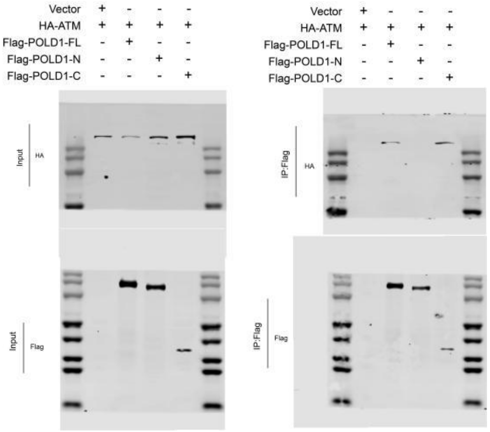

Fig6 H

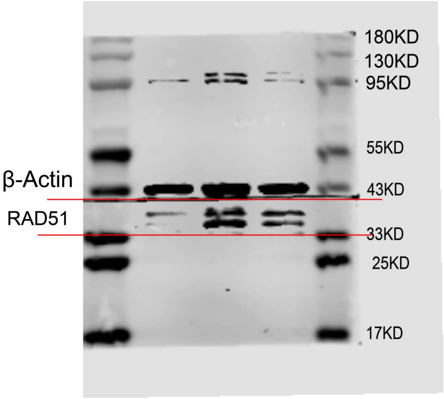

Figure7 C

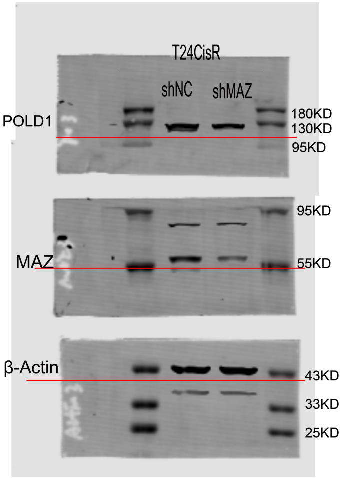

β-Actin 100 : 103
MAZ 100 : 48
POLD1 100 : 71

Figure7 E

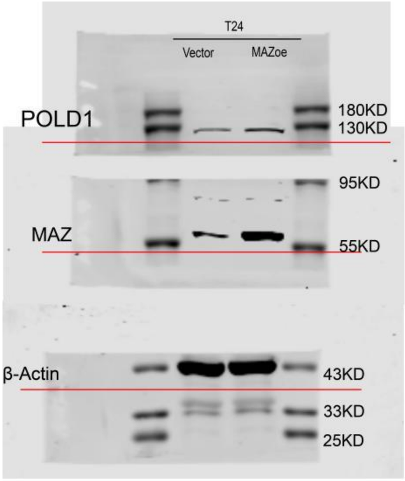

β-Actin 100 : 101
MAZ 100 : 305
POLD1 100 : 189

Figure8 A

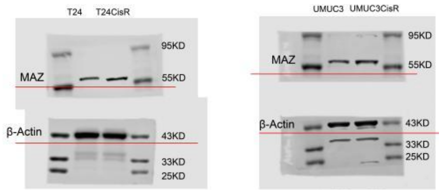

β-Actin 100 : 91
MAZ 100 : 137

β-Actin 100 : 102
MAZ 100 : 139

Figure8 D

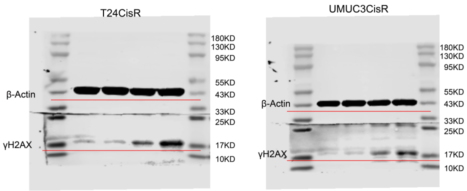

β-Actin 100 : 106 :97: 103
γH2AX 100 : 82 :258: 575

β-Actin 100 : 104 :96: 108
γH2AX 100 : 124 :265: 467

Figure8G

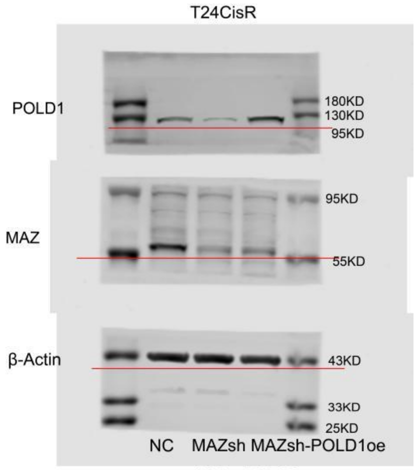

β-Actin 100 : 100:92
MAZ 100 : 47 :52
POLD1 100 : 25 :155

Figure8 I

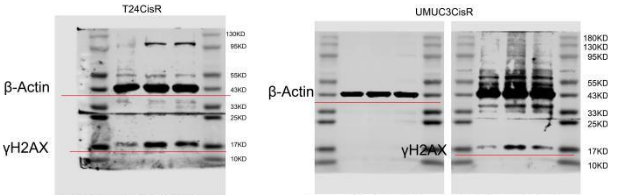

β-Actin 100 : 104:101
γH2AX 100 : 228 :176

β-Actin 100 : 106:104
γH2AX 100 : 481 :249

FigureS2A

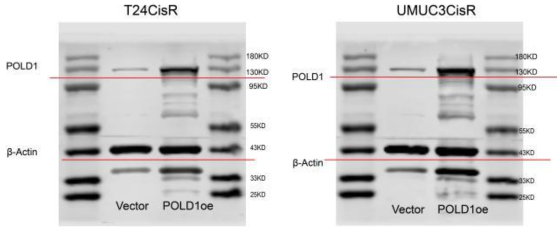

β-Actin 100 : 107
POLD1 100 : 975

β-Actin 100 : 106
POLD1 100 : 529

FigureS2 H

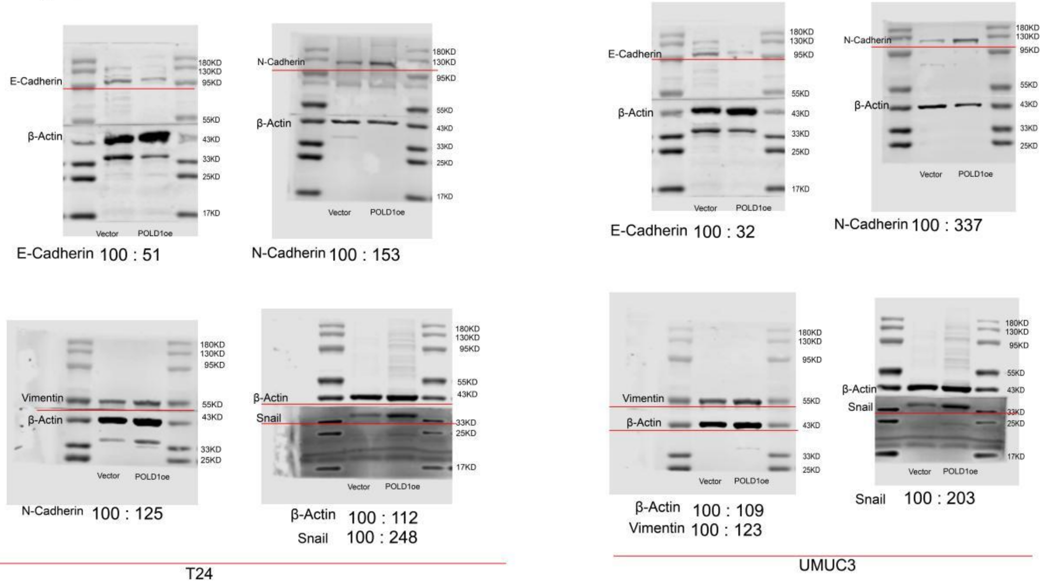

FigureS4B

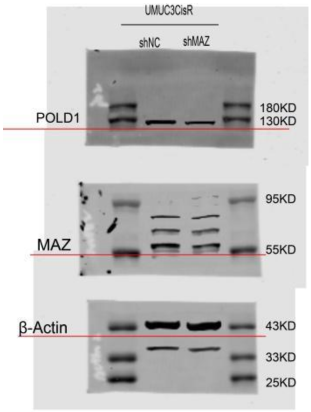

β-Actin 100 : 103
MAZ 100 : 64
POLD1 100 : 68

FigureS4D

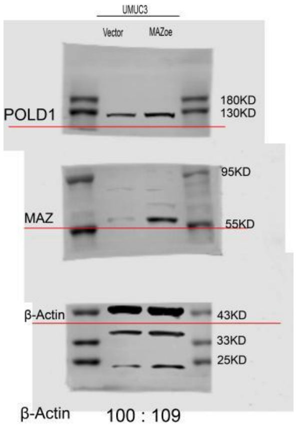

β-Actin 100 : 109
MAZ 100 : 596
POLD1 100 : 158

FigureS4F

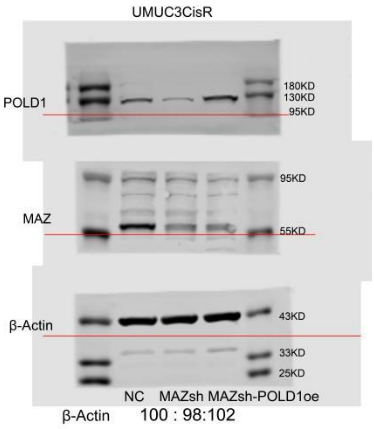

β-Actin 100 : 98:102
MAZ 100 : 51 :43
POLD1 100 : 23 :159
